# ^210^Pb and ^137^Cs dating models as tracers of recent sedimentary processes of the shallow lake under anthropogenic activity

**DOI:** 10.1038/s41598-025-31649-z

**Published:** 2026-03-28

**Authors:** Noha Imam, Alia Ghanem, Afaf Nada, Hosnia Abu-Zeid, Said A. Shetaia, Waiel E. Madcour

**Affiliations:** 1 Physics & Geology Lab, National Institute of Oceanography and Fisheries (NIOF), Cairo, Egypt; 2 Physics Department, Faculty of Women for Arts, Science & Education, Ain Shams University, Cairo, Egypt; 3 Geology Department, Faculty of Science, Al-Azhar University, Cairo, Egypt; 4 Radiation Protection Department, Nuclear Research Center, Egyptian Atomic Energy Authority, Cairo, Egypt

**Keywords:** Radiometric dating, INAA- k_0_ method, Edku lake, Accumulation rate, Depositional metal fluxes (DMF), Biogeochemistry, Environmental sciences

## Abstract

One of Egypt’s most vulnerable aquatic wetlands is Lake Edku, the third-largest lake in the Nile Delta. Many wastes are found in the water due to anthropogenic activities and contamination of nearby cities, fish farms, and agricultural areas, particularly through drains, which result in several ecological issues. Despite this, sedimentary processes are used as archives, providing a unique opportunity to document the changes the environment has undergone over the past 100 years. To study the sedimentation and age of Lake Edku, this study employed a high-purity Germanium gamma spectroscopy (HPGe) system to create a chronological sequence of four sedimentary cores based on the analysis of ^210^Pb_ex_ and ^137^Cs. Determining element concentrations was also done at the Second Research Reactor (ETRR-2) in Egypt using instrumented neutron activation analysis (INAA-k_0_ technique). Several dating models for ^210^Pb and ^137^Cs were employed. According to the study, the constant rate of supply (CRS) model proved to be the most reliable for dating sediment cores, exhibiting a high degree of agreement with the chronology determined by the ^137^Cs approach. The direct release of massive volumes of industrial and home wastewater is one of the most likely anthropogenic inputs that have contributed to increased silt deposition, particularly during the 1990s. The vertical profile of metals reveals the history of metal deposition, making it easier to understand the characteristics of accumulation in various sedimentary layers. The amount of heavy metals in sediments is influenced by the rate of sedimentation at the sampling site, as well as the emission from sources due to sediment dilution. Because sedimentation rates vary so much, the anthropogenic contribution is reflected far more effectively by the metals deposition flux (DMF) than by their concentrations. Overall, the post-dam transformation of Lake Edku demonstrates a shift from Nile-controlled sedimentation to a drainage-dominated system, resulting in accelerated sedimentation rates, nutrient enrichment, and severe ecological deterioration.

**Supplementary Information:**

The online version contains supplementary material available at 10.1038/s41598-025-31649-z.

 Urbanization and rapid industrialization have made environmental issues in coastal and estuarine regions worse^1^. The coastal lakes, which are made up of quite deep and semi-enclosed water bodies^2^, have significant cultural, ecological, and aesthetic value as vital ecosystems^3^. A variety of human activities, including industrialization, aquaculture, maritime transportation, and tourism, greatly expose these lakes, resulting in a variety of pollutants^4^. Throughout the industrial revolution, human activities have increased significantly, contributing to the serious environmental contamination in these lagunes^5^. Many industrial, agricultural, and household wastes, as well as domestic waste and sewage, are dumped into these lagoons, posing a serious threat to their sustainability. It is acknowledged that heavy metals are among the most concerning environmental pollutants in aquatic environments due to their toxicity, persistence, and lack of biodegradability^6^. The heavy metals found in aquatic systems originate from both natural sources (primarily erosion and erosion of the rocky soil) and human activities (such as industrial waste, agricultural activities, sewage disposal, and wastewater runoff)^2^. Understanding the origins and examining the historical development of these heavy metals are crucial for managing the risks associated with metal pollution and implementing mitigation strategies.

The lake sediments act as a record of environmental changes and may contain a variety of contaminants and elements that are naturally occurring or artificially caused^7,8^. The chronological framework of contamination represented by the sampled strata is an important component of sedimentary records^9,10^. The study of radiotracers in marine sediments has greatly contributed to our understanding of recent contamination profiles as well as environmental changes. Radiotracers like cesium-137 (^137^Cs), which has a half-life of T_1/2_ = 30.05 (8) years, and excess lead-210 (^210^Pb_ex_), which has a half-life of T_1/2_ = 22.23 (12) years^11^, have been used extensively for dating and tracking contemporary sediments as well as assessing the level of contamination in marine environments. Therefore, extrapolated sedimentation rates show changes in the depositional environment and take into account the previous accumulation profile of metals^12^. This enables the effectiveness of environmental policies to be examined. Another crucial consideration is assessing the effects of long-term pollutant release on coastal and marine ecosystems, in addition to pollution history. The degree of sediment contamination by heavy metals from man-made sources and the possible ecological risk assessment are commonly determined by the following indices: the enrichment factor (EF) and the depositional metal flux (DMF)^4^, respectively.

All environmental compartments contain the natural radionuclide ^210^Pb, which has been used as an environmental radiotracer for a variety of processes, including sedimentation processes^13–15^, ocean biogeochemistry, atmospheric deposition and contamination^16^, evaluating the recent effects of microplastics^17,18^, and developing age-depth models^19^ that look at changes during a period of growing anthropogenic changes to ecosystems. The radioisotope ^210^Pb belongs to the ^238^U natural radioactive series^20^ and is a daughter product of ^222^Rn (T_1/2_ = 3.8 days). Two components make up ^210^Pb dating in sedimentary processes: (i) excess ^210^Pb (^210^Pb_ex_) from the radioactive decay of ^222^Rn in the atmosphere and water column^21^ and (ii) supported ^210^Pb (^210^Pb_sup_) from the radioactive decay of ^226^Ra (T_1/2_ = 1600 ± 7 years). According to the usual radioactive decay rule^22^, the ^210^Pb dating method shows that the ^210^Pb_ex_ activity decreases with age in each sediment layer. Many long-lasting chronologies for sedimentation rates and pollution loadings have been established using this radionuclide, spanning a dating horizon of roughly seven half-lives, or some 120 to 150 years^8^. Additionally, in this context, the man-made radionuclide ^137^Cs is a known chronostratigraphic marker that identifies the support horizon from distinct and easily identifiable atmospheric sources, especially the nuclear weapons tests of the 1950 s and the 1960 s, the peak in 1963 was particularly noteworthy^7^. During those years, weapons test fallout records were used to calculate the depth of sedimentary layers. The Chernobyl explosion in April 1986 caused a ^137^Cs surge across Europe and the Mediterranean region, which is another important time marker. The ^137^Cs peak brought on by the Fukushima Daiichi nuclear accident (Japan, March 2011) is another significant factor^23,24^. Recently, the preferred method for establishing the chronologies of recent sediment profiles has been the combination of ^210^Pb_ex_ and ^137^Cs^25,26^.

Three models based on ^210^Pb—the model of constant flux and constant sedimentation (CF-CS), the model of constant initial concentration (CIC), and the model of constant rate of supply (CRS)—can be used to reconstruct sediment profile sequences. The sedimentation processes that take place in all aquatic ecosystems must be considered when choosing a model. This occurs because each model bases its hypotheses on particular environmental conditions. One significant assumption made by all of these models is that ^210^Pb^10^ does not have post-depositional mobility. The simplest model is constant flux-constant sedimentation (CFCS)^27^. Taking into account a constant ^210^Pb_ex_ flux and sedimentation, the activity of ^210^Pb_ex_ decreases significantly with the accumulation of dry mass (that is, a linear line on a semi-logarithmic scale)^28^. The models of CRS and CIC are used for variable sedimentation rates^16,29,30^. According to the CIC model, which assumes a constant initial concentration of ^210^Pb_ex_ at the water–sediment interface, the ^210^Pb_ex_ supply rate is proportional to the sedimentation rate^31^. To apply the CIC model, the unsupported ^210^Pb activity must show a monotonic drop with depth^32^. Using the Constant Rate of Supply (CRS) model, the age of a given depth is determined from a ^210^Pb vertical profile in a sediment column. It is predicated on three assumptions: that the starting concentration of ^210^Pb in sediment is changeable, that the sediment influx rate is variable, and that unsupported ^210^Pb is delivered to sediments at a consistent rate over time^33^. Only the CRS model can be used to explain the stratigraphic chronology when the ^210^Pb activity-to-depth profile is nonlinear and very nonmonotonic^34^. To lower the uncertainty in the interpretation of its distribution, each ^210^Pb_ex_ geochronology needs to be verified with at least one independent tracer^35,36^.

The Nile Delta is located in the arid climate zone with an average annual rainfall of only about 100 mm (Fig. 1). However, the Nile Delta is home of > 40% of Egyptians who are engaged in agriculture and industry, in addition to four coastal lagoons^37^. The total wetland area of the four lagoons is ca. 2,400 km2, accounting for ca. 10% of the delta area, a key area of Egypt’s socio-economic and ecological system^37,38^. The four lagoons are also the natural fishing grounds, which account for 50% of Egypt’s fish production^39^. Due to intensifying agricultural and industrial activities in adaptation to increasing population (Fig. 1), the water bodies of the delta (lagoons and distributaries) have received a huge amount of agricultural and industrial pollutants through the dense network of drains (Fig. 1), which leads to the deterioration of the ecosystem^38,40–42^. The total amount of sewage water derived from industrial, agricultural, and domestic sources reaches > 16 billion m3/yr, which is discharged into the four lagoons (Manzala, Burullus, Edku and Mariut) at the front of the Nile Delta through the networks of artificial and natural canals widely across the delta plain^38,43,44^. Notably, because of the decreased fluvial dynamics brought on by the Aswan High Dam, the Nile lagoons serve as the last reservoir for most sewage, including industrial and agricultural effluent^38^. Mariout lagoon contains the effluents of Alexandria’s industries, whereas Manzala lagoon receives the majority of Cairo’s wastewater as well as the eastern delta’s municipal, agricultural, and industrial effluents^40^. Additionally, the central delta’s Burullus lagoon receives around 950 × 10^3 a-1 of wastewater from megacities and agricultural areas (such as Tanta, Kafr El-Shaikh, and El-Mahalla)^45,46^. Consequently, especially following the Aswan High Dam, the concentration of heavy metals in the lagoon sediments has grown over time^40,46,47^.

Moreover, lake Edku has been polluted by many sources since the early 1990 s, and this is still happening in the twenty-first century^48^. Lake pollution comes from a variety of point- and non-point sources, such as nearby municipal and agricultural wastewater^49^. Because untreated sewage pollution has reduced the lake area, there have been significant economic repercussions, particularly on fisheries^50^. Rapidly declining lake conditions pose a serious threat to the lake’s continued existence. Previous research establishes that Lake Edku suffers from severe environmental degradation, driven primarily by pollution from agricultural, domestic, and industrial discharges (Fig. 1). Studies confirm the lake is hyper-eutrophic^51,52^ and contaminated with heavy metals exceeding safe limits in water, sediment and fish tissue^53,54^. This pollution has caused significant ecological damage, evidenced by oxidative stress in fish^55^ and deformed, low-diversity benthic foraminiferal communities^56^, culminating in tangible human health risks from fish consumption^53^. Collectively, these findings underscore an urgent need for improved management and conservation efforts for this vital lagoon ecosystem^57^.

Introduction This study aims to investigate the causes of pollution and the processes that lead to wetland degradation by analyzing the temporal and geographical distribution of heavy metals in the delta area. Using sediment core samples from Lake Edku, the study will investigate pollution indices, historical trends, and fluxes of human activities, as well as sediment chronology and sedimentation rates. By protecting the environment and reducing pollution levels, this research will inform decisions regarding the future distribution and structure of Lake Edku.

## Methodology

### Study area

Edku Lake, located in Egypt’s northern delta, is regarded as one of the country’s most prominent lagoons. The origin of the delta’s lagoons is due to Nile floodwaters trapped behind coastal sand dunes in the low-lying delta; they are not maritime in origin^37^. Starting around 6500 years ago, sand ridges formed along the delta’s edge, acting as a natural barrier that allowed marshes and lagoons to develop^58^. Lake Edku originated as a shallow, brackish-to-freshwater coastal lagoon formed behind a series of sand bars and dunes along Abu Qir Bay. Historically, it was maintained by a delicate balance between freshwater inflows from the Nile distributaries and intermittent incursions of Mediterranean seawater through the El-Boughaz outlet^59,60^. Prior to 1970, the lake’s basal sediments consisted mainly of grayish Nile silt—fine-grained clay rich in montmorillonite and illite—deposited during the annual Nile floods^40,61^. These sediments were highly fertile, enriched with bulk and trace elements such as Fe, Mg, K, and P. However, the construction of the Aswan High Dam (AHD) in the late 1960 s cut off the natural Nile sediment supply, transforming Lake Idku from a dynamic deltaic lagoon into a drainage and wastewater retention basin dominated by agricultural and municipal inputs^62^. This shift led to rapid sediment infilling, a marked reduction in the lake’s surface area, and severe ecological degradation^63^. The modern sediment layer is hyper-eutrophic, characterized by elevated organic matter and contamination with heavy metals such as Pb, Cd, and Zn derived from agricultural runoff and industrial effluents^48,51^.

**Fig. 1:**
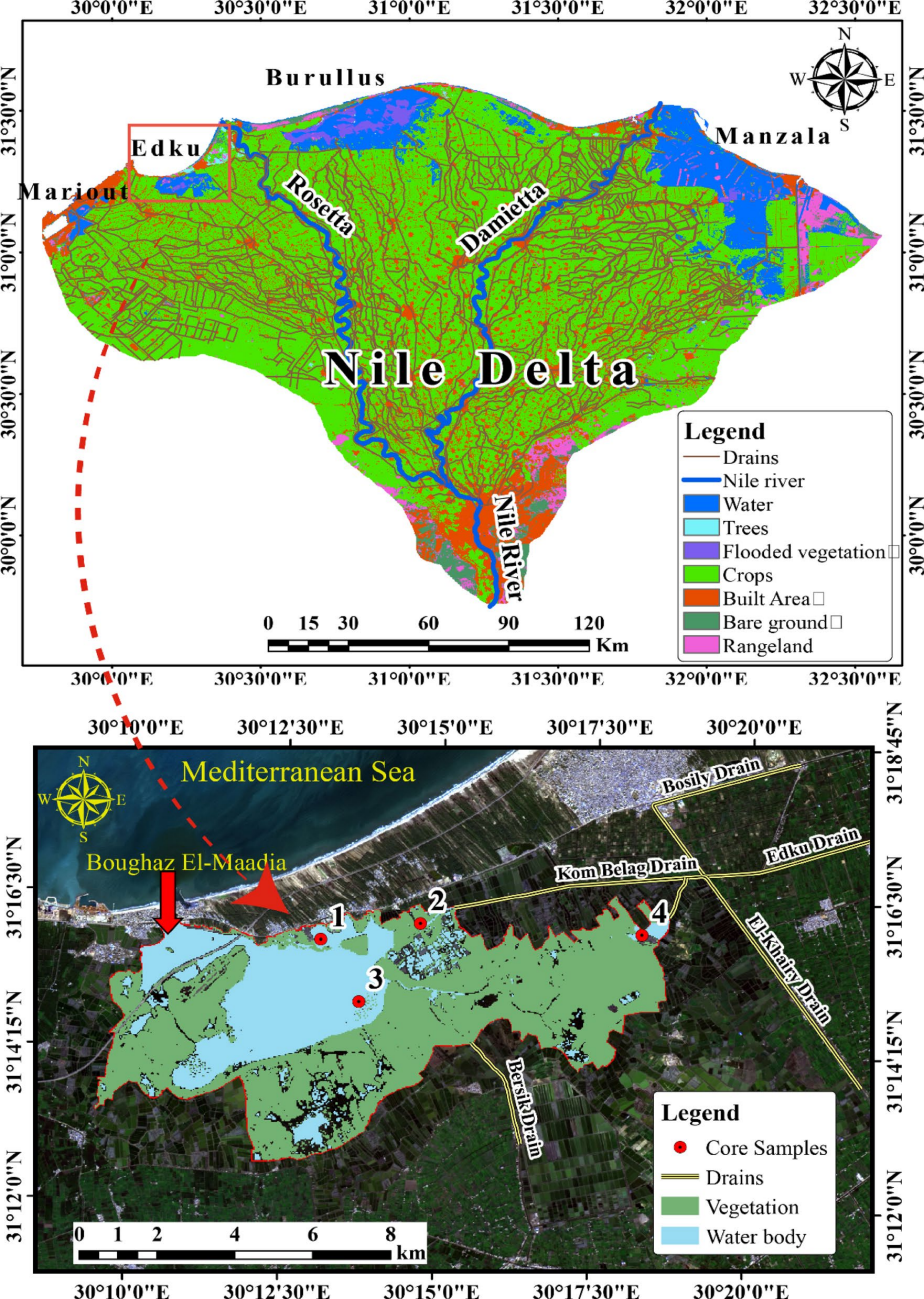
Map of Edku Lake and sampling sites using ArcGIS 10.8 ^®^ software by Esri. ArcGIS^®^ and ArcMapTM are the intellectual property of Esri. Copyright © Esri. (All right https://www.esri.com/en-us/home*)*.

It is located in the Beheira Governorate, 30 km from the Abu Qir coast of the Alexandria Governorate, between longitudes 30° 08′ 30″ and 30° 23′ E and latitudes 31° 10′ and 31° 18′ N (Fig. 1). Moreover, the lake is connected to the Mediterranean Sea via Boughaz El Maadia. Between 2000 and 2018, the Edku Lake region changed significantly, with large areas being reclaimed for agriculture^49^. The lake has a surface area of about 62.7 km², of which 22 km² is open water and the rest is made up of islands and aquatic vegetation^63^. The highest depth of this lake is about two meters, making it incredibly shallow (see Sheta et al.^64^). The salinity of its brackish water ranges from 2.5 to 15 g/L in the vicinity of northern inlet^46^. Valuable farmland is located to the south of the lake, current reclamation projects are located to the east, while residential neighbourhoods and factories are located to the west. Since the 1800 s, this region has undergone significant reclamation^65^. ElKhayry and Barsik are the two primary drains that discharge waste into Lake^66^. The first drain connects three sources of drainage: El-Bosely, Edku, and Damanhour. These subdrains transport home, agricultural, and industrial effluent, as well as water from over 300 fish farms. The second drain is mostly utilized to discharge agricultural drainage water into the lake. Agricultural runoff contributes 2.62 million m³ to the lagoon, negatively impacting its chemical quality^67^.

The lake’s sedimentary regime underwent a fundamental transformation following the construction of the Aswan High Dam, which effectively cut off the annual supply of natural, fluvial sediment from the Nile River. The catchment now primarily drives sedimentation via annual discharges of drainage water from major drains, such as Kom Belag and Bersik, which serve as the lake’s main water source^59,60^. This drainage carries an excessive nutrient and pollutant load^51^, leading to both: (1) high rates of biogenic organic sediment accumulation due to hyper-eutrophication; and (2) the concentration of fine-grained, contaminated sediments^48^. Compounding these effects, intensive land reclamation and changing land use in the surrounding catchment have caused a significant diminution of the lake’s open water area, which in turn concentrates the incoming sediment load and rapidly accelerates the overall accretion and infilling rate within the remaining basin^60,63^.

### Sampling and samples preparation

The four core sediments were picked up from Lake Edku with a Global Positioning System (GPS), as shown in Table 1 and Fig. 1. The sampling locations were chosen based on the amount of water mixing and circulation, the lake’s irregular shape, and the presence of drains, canals, and connections to the Mediterranean Sea. The maps were made using ArcGIS 10.8 software by Esri. ArcGIS and ArcMapTM are the intellectual property of Esri and are used here under license. Copyright © Esri. All rights reserved. For more information about Esri^®^ software, please visit (https://www.esri.com/en-us/home*).* The sediment samples were taken manually by inserting a sediment core with a diameter of 10 cm. A tight pneumatic seal was used to lift the core samples at the top of the tube. As soon as the core was extracted, both ends were sealed with plugs, and it was taken to the lab for further examination. Until they were ready for analysis, the samples were kept in a refrigerator at 4 °C in the laboratory. The four cores were cut to a depth of 6 cm, dried in an oven at 60 °C, fragmented, and then crushed with a mortar and pestle before being sieved through a 125-mesh sieve. The samples were put into 100-mL Marinelli beakers.

**Table 1:** Location of sampling sites of Edku lake.

Lake	Core profile	Coordination		Length of sediment core cm
		Longitude	Latitude	
Edku	ED-1	30° 13’3.54"E	31° 15’50.783"N	72
	ED-2	30° 14’40.223"E	31° 16’3.918"N	72
	ED-3	30° 13’41.297"E	31° 14’59.363"N	66
	ED-4	30° 18’14.286"E	31° 16’2.434"N	60

### ^210^Pb_ex_ dating methods

In order to quantify sedimentation rates based on the ^210^Pb_ex_ depth profiles in this work, a chronological sequence or depth of age for the core must be constructed using a model. Three models have been extensively and effectively implemented: the CIC, CRS, and CF-CS models.

The CF-CS model assumes a constant mass accumulation rate and a constant flux to the sediment surface^33^, which are represented as follows:

1 $$\:{C}_{i}={C}_{0}{e}^{-\lambda\:{m}_{i}/R}$$

C_i_ and C_0_ (Bq kg^–1^) represent ^210^Pb_ex_ activity at the interface of water and sediment, R represents mass accumulation rate (g cm^–2^ yr^–1^), and m_i_ represents total mass depth to the i^th^ layer (g cm^–2^).

To determine mass accumulation rates, the following equation is calculated using linear regression between the C_i_ and the mass depth^31^:

2 $$\:\mathrm{ln}{C}_{i}=\mathrm{ln}{C}_{0}-\lambda\:{m}_{i}/R$$

The slope of the linear regression formula (y = a + bx) is b = -λ/R, which implies that $\:R$ = -λ/b. As a result, the layer age *T*_*i*_ is given as follows:

3 $$\:{T}_{i}={m}_{i}/R$$

The CIC model states that the initial ^210^Pb concentration in deposited sediment is constant regardless of the rate at which sediment accumulates^68^. This hypothesis suggests that when sedimentation occurs, the concentration of ^210^Pb_ex_ is constant and has a C_0_ value. Based on this hypothesis, sediment age $\:{T}_{i}\left(yr\right)$ can be written as follows:

4 $$\:{T}_{i}\left(yr\right)=\left(1/\lambda\:\right)\mathrm{ln}\left({C}_{0}/{C}_{i}\right)\:$$

The CRS model assumes that the ^210^Pb_ex_ (f) flux is constant while the sedimentation rate fluctuates over time^27^. As a result, this procedure is used in various ecosystems where sediment may vary due to climatic or human influences^8^. The CRS model allows for the calculation of Layer (*T*_*i*_) at i, MAR (gm cm^–2^ yr^–1^), and SAR (cm yr^–2^) using the following equations^32,69^.

5 $$T\left[ {\left( i \right),yr} \right] = ~\frac{1}{\lambda }\ln \frac{{I(0)}}{{I(i)}}$$

6 $$MAR~ = \frac{{\lambda ~I(i)}}{{C\left( i \right)}}$$

7 $$\:SAR\:=\frac{MAR}{\rho\:\left(i\right)}*100$$

where *I(0)* (Bq m^− 2^) is the total ^210^Pb_ex_ inventory of the entire sediment core, *I(i)* (Bq m^− 2^) is the total ^210^Pb_ex_ inventory in the sediment core below depth i, C(i) is the ^210^Pb_ex_ activity in layer (i) and $\:{\uprho\:}\left(\mathrm{i}\right)$ is the mean dry bulk density of a certain layer.

The total inventory of ^210^Pb_ex_ beneath layer (i) is given by:

8 $$\:I\left(i\right)=\sum\:_{j=i+1}^{j=\infty\:}\varDelta\:{I}_{j}$$

$\:\varDelta\:{I}_{j}$ is the accumulation of ^210^Pb_ex_ deposits below section (i) and given by :

9 $$\:\varDelta\:{I}_{i}=\:{C}_{i}[\varDelta\:{m}_{i}/S]$$

Where S is the core cross-section and *∆m*_*i*_ is the dry weight of section i (kg).

The uncertainty in the computed ages and sedimentation rates of the CFCS, CIC, and CRS models is calculated using formulae from^16,32^.

### The enrichment factor (EF)

The enrichment factor is a quantitative metric used to quantify pollution and determine the distribution of anthropogenic elements in sediments^70^. To account for particle size variations, the sample’s elemental concentration was standardized with Al as a reference element^71^.

EF values can be calculated as follows^72^:

10 $$\:\mathrm{E}\mathrm{F}=\raisebox{1ex}{${(\mathrm{M}\mathrm{e}\mathrm{t}\mathrm{a}\mathrm{l}/\mathrm{A}\mathrm{l})}_{\mathrm{S}\mathrm{a}\mathrm{m}\mathrm{p}\mathrm{l}\mathrm{e}}$}\!\left/\:\!\raisebox{-1ex}{${(\mathrm{M}\mathrm{e}\mathrm{t}\mathrm{a}\mathrm{l}/\mathrm{A}\mathrm{l})}_{\mathrm{b}\mathrm{a}\mathrm{s}\mathrm{e}\mathrm{l}\mathrm{i}\mathrm{n}\mathrm{e}}$}\right.$$

where (Metal/Al)_Sample_ and (Metal/Al)_baseline_ represent the ratios of metal to Al in sample and baseline reference element concentrations^73^, respectively.

### Depositional metal flux (DMF)

The following equation was used to calculate depositional metal fluxes (DMF, µg/cm²/yr) for each core section, utilizing metal concentration (M, mg/g) and MAR (gm/cm²/yr).

11 $$\:DMF=\:MAR\times\:M$$

### Analytical techniques

#### Radionuclides determination by HPGe detector

The four sediment core samples were analyzed for ^210^Pb, ^226^Ra, and ^137^Cs by gamma spectrometry using two hyper-purity germanium (HPGe) detectors (N-type [model GMP-100 250-S at the Inshas Nuclear Centre of the EAEA has a relative efficiency of 100%] and P-type [has a relative efficiency of about 50% when compared to the 3ʺ × 3ʺ efficiency of the crystal NaI(Tl)]). The spectra were evaluated with the computer software program Maestro (EG&G ORTEC) and manually with the use of a spreadsheet (Microsoft Excel 365) to calculate the natural radioactivity. The detector’s efficiency was calibrated by measuring the spectrum of a source that emits gamma rays with known energy, using the IAEA-410 standard sources RGU-1, RGTh-1, RGK-1 (IAEA 1987). The activity concentration of radionuclides ^210^Pb, ^137^Cs, and ^226^Ra (^214^Pb and ^214^Bi) is expressed as Bq/kg for sediment. The counting time has been set to 72,000 s in order to obtain a good statistical result for the background and sediment samples. The activity of ^210^Pb_total_ was calculated based on the photopeak at 46.5 keV and corrected for self-absorption^32^. The correction factor was determined by measuring a point source of ^241^Am placed above the container filled with sediments and the empty container, following the procedure used by^74^. The activity concentrations of ^226^Ra were estimated using its progeny (^214^Pb and ^214^Bi), which had energies of 295.1 keV and 352.0 keV, as well as 609.3 keV and 1120.3 keV, respectively. The excess ^210^Pb activities are calculated by subtracting the actual ^226^Ra activity from the total ^210^Pb activity. The activity of ^137^Cs was measured directly using the gamma line at 661.7 keV. The activity concentration of sediment samples (A_S_) can be determined using the following equations^75^.

12 $$\:{A}_{S}=\frac{CPS\:}{{P}_{\gamma\:}\times\:\epsilon\:\times\:w\left(kg\right)}$$

Where *CPS* is the counting rate under each photopeak, $\:{P}_{\gamma\:}$ corresponds to the probability of emitting each gamma ray, **ε** is the efficiency index (which varies depending on energy), and w represents the sedimentary sample mass (kg). The effectiveness of the detector, geometry, counting statistics, and the intensity of the gamma radiation are all important factors that determine the concentration of gamma radiation in environmental samples. To calculate the uncertainty of activity concentration (*U*_*A*_), use the formula below^32,76–78^:

13 $$\:{\:U}_{A}=A\times\:{\left(\frac{U\left(CPS\right)}{CPS}+\frac{U\left({P}_{\gamma\:}\right)}{{P}_{\gamma\:}}+\frac{U\left(\epsilon\:\right)}{\epsilon\:}+\frac{U\left(w\right)}{w}\right)}^{1/2}$$

where $\:U\left(CPS\right)$, $\:U\left({P}_{\gamma\:}\right),\:U\left(\epsilon\:\right),\:U\left(w\right)\:\mathrm{c}\mathrm{o}\mathrm{u}\mathrm{n}\mathrm{t}\:\mathrm{r}\mathrm{a}\mathrm{t}\mathrm{e}$, emission probability of each gamma-ray, efficiency and sample mass uncertainties, respectively. The statistical uncertainty was approximately 10% over the whole energy range.

#### INAA elemental analysis

Twelve samples from two sediment cores, ED-2 and ED-3, were irradiated in the Egypt Second Research Reactor’s (ETRR-2) neutron activation analysis lab, which primarily produces thermal neutrons. The samples undergo crushing, homogenization, and sieving through 125 mesh sieves. About 100–300 mg of the samples was enclosed in a high-purity polyethylene capsule for long and short radiation. For short radiation exposures, a pneumatic transport system was used to move polyethylene capsules with a sample to an irradiation position. The samples were exposed to a thermal neutron flux of 4 × 10^11^ cm^− 2^ s^− 1^ for 30–60 s using a pneumatic rabbit system. For long irradiation, samples were placed in aluminum recipients and subjected to a thermal neutron flux of 1.5 × 10^14^ cm^− 2^ s^− 1^ for 1 h. Aluminum can hold 12 high-purity polyethylene capsules, a blank polyethylene receiver, and a flux monitor set (Au, Zr).

Gamma ray measurements were carried out using a gamma spectrometer equipped with an HPGe detector. EG&G Ortec provided all of the system’s components. The detector’s energy and efficiency were calibrated using Isotope Products Laboratories’ ^133^Ba, ^137^Cs, ^60^Co, and ^152^Eu point sources.

The concentration of the element ($\:{C}_{a},ppm$) under investigation can be calculated as^79^:

14 $$\:{C}_{a}=\frac{{\left[{A}_{sp}\right]}^{a}}{{\left[{A}_{sp}\right]}^{*}}\times\:\frac{1}{{k}_{0,m}\left(a\right)}\times\:\frac{f+{Q}_{0}^{*}\left(\alpha\:\right)}{f+{Q}_{0}^{a}\left(\alpha\:\right)}\times\:\frac{{\epsilon\:}_{p}^{*}}{{\epsilon\:}_{p}^{a}}\:\times\:{10}^{6}$$

Here, $\:{C}_{a}$ is the element’s concentration (µg/g), and $\:{A}_{sp}$=$\:\:\frac{{N}_{p}/{t}_{m}}{W\left(1-{e}^{\lambda\:{t}_{i}}\right){\:e}^{-\lambda\:{t}_{d}}\:\left(1-{e}^{\lambda\:{t}_{m}}\right)}$ represents the specific activity of the sample element (a) and the ^198^Au comparator (*), where $\:{N}_{p}$ is the net peak area, $\:{t}_{d}$ is the decay time, $\:{t}_{i\:}$is the irradiation time, $\:{t}_{m}$ is the measuring time, W is the weight, and $\:\lambda\:=(1/{T}_{1/2})\:$is the decay constsant. $\:{Q}_{0}\left(\alpha\:\right)$ indicates the resonance integral to the 2200 m/sec cross-section ratio, whereas *f* represents the thermal-epithermal neutron flux ratio at the irradiation site in the reactor. The distribution of epithermal neutron flux, denoted by α, can be approximated by a $\:1/{E}^{\alpha\:+1}$ dependency, independent of neutron energy. The detection efficiency at each gamma energy of the sample element and 198Au comparator is denoted by $\:{\epsilon\:}_{p}$. The $\:{k}_{0}$ factor of a comparator is represented by $\:{k}_{0,m}\left(a\right)=\:\frac{{k}_{0}^{*}\left(a\right)}{{k}_{0}^{*}\left(m\right)}$. The uncertainty in the concentration of the element is estimated using the equation from^32^.

### Quality assurance

The National Institute of Oceanography and Fisheries in Cairo, Egypt, supplied the reference materials IAEA-412 and IAEA-312 (soil), which were used to determine the accuracy and precision of the radionuclide measurements. For INAA, the IAEA-Soil 7 reference was used to determine elemental concentrations, confirm them, and assess the accuracy of the used method. For the majority of elements and radionuclides, the precision was higher than 10%.

### Statistical analyses

Statistical analysis was performed on the analytical data concerning the distribution and correlation between the parameters of the investigation. Microsoft Excel (365) and Minitab are essential tools for statistical analysis. The Pearson correlation matrix, which is based on Microsoft Excel (365), is used to determine the origin of heavy metals (HMs). However, several methods are based on the principal component analysis test (PCA) to determine the elements present in the core sediments^80–82^. PCA is a tool used to analyze HM sources, which is performed by the Canoco-5 program.

## Results and discussions

### Depth profile of radionuclides

**Fig. 2:**
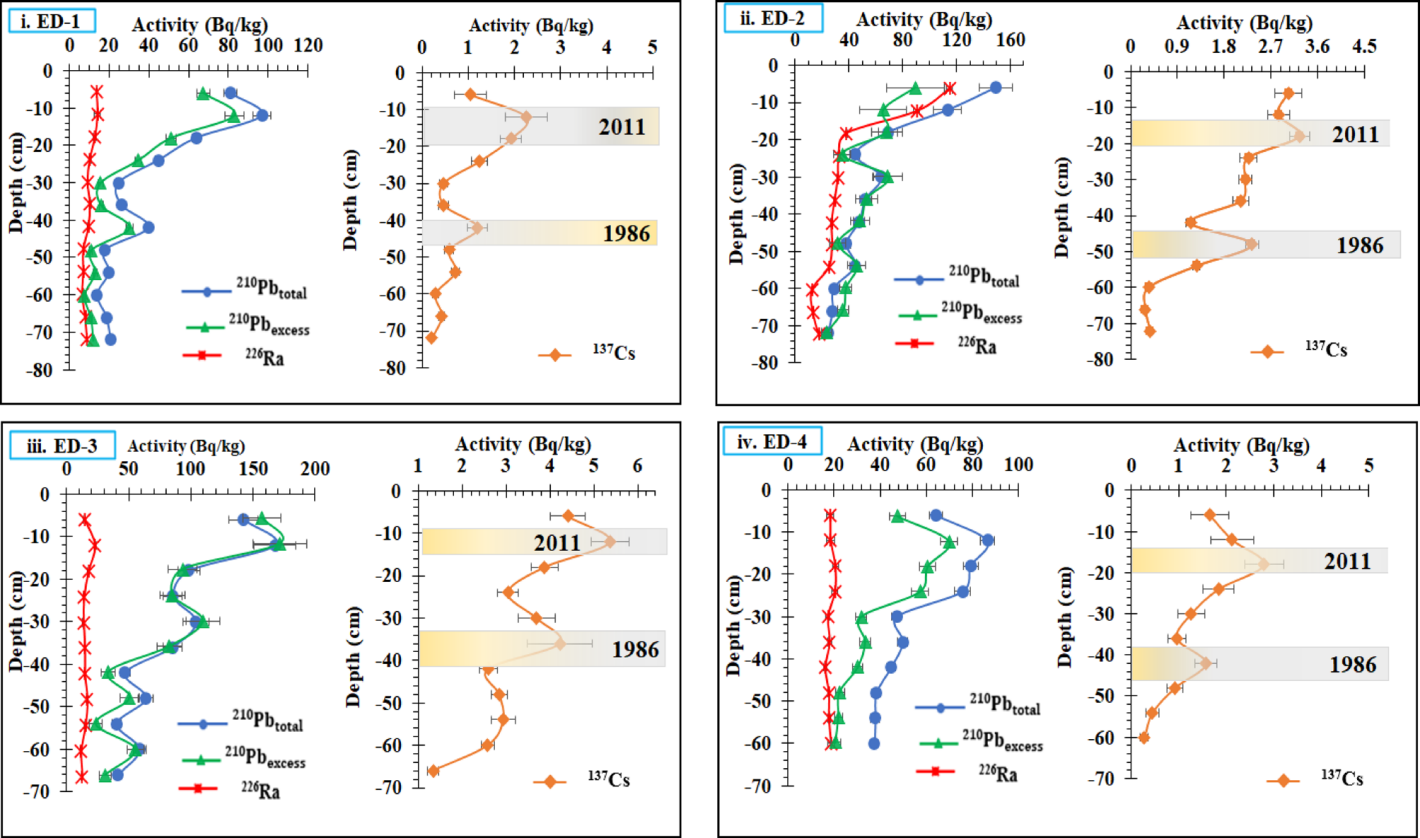
Depth profiles ^210^Pb_Tot_, ^226^Ra, ^210^Pb_ex_, and^137^Cs in sediment core samples of Edku Lake.

The vertical distributions of ^210^Pb_Tot_, ^226^Ra, ^210^Pb_ex_, ^137^Cs, ^232^Th, and ^40^K activity in the four sediment cores are shown in Fig. 2 and Table 2. ^210^Pb_Tot_ activity decreased almost exponentially with depth in all Lake Edku sediment cores. The ^226^Ra activity concentrations were nearly constant in each core, with an average value of 10.12 ± 0.50 Bq/kg (core ED-1), 31.55 ± 0.80 Bq/kg (core ED-2), 23.14 ± 0.91 Bq/kg (core ED-3), and 17.53 ± 1.16 Bq/kg. The distribution of ^210^Pb_ex_ activity was similar to that of ^210^Pb_Tot_ activity, which can be explained by the fairly constant ^226^Ra activity. The activity of ^210^Pb_ex_ was calculated for each sample, subtracting the activity of ^226^Ra from the activity of ^210^Pb_Tot_^83^. In ED-2, the depth distributions of ^210^Pb_ex_ decreased significantly as the depth increased. This allowed us to calculate sedimentation rates for each core and use them to study both human and natural activities that affect sediment flow. There are possible explanations for the anomalies in ^210^Pb_ex_ activities at locations ED-1, ED-3, and ED-4. First, it is most likely due to temporal variations in ^210^Pb_ex_ fluxes linked with sedimentation, which were caused by considerable changes in land use and vegetation cover throughout the 1920s^84^. Second, vertical sediment mixing caused by physical and/or biological activity in the sediment column is likely to have an impact on the detected ^210^Pb_ex_ activity profile. This is due mostly to the presence of large-scale mariculture operations in the area^10^. Frequently occurring fishing and mariculture activities may significantly impact the sediment, which can lead to vertical remixing. Furthermore, a vertical redistribution of sediments may be the consequence of some bioturbation. However, other processes can have similar effects on sediment records; for instance, reduced ^210^Pb_ex_ activities at a core surface segment (e.g. cores ED-1, ED-3, and ED-4) could be the result of very rapid sedimentation^85^. As shown by^27^, ^210^Pb_ex_ activities in the sediments result from the balance between the ^210^Pb_ex_ flux to the sediments and the sediment load (i.e. mass accumulation rate, MAR); this implies that ^210^Pb_ex_ activities would decrease as a result of dilution by a higher MAR, or increase if the MAR was lower. In addition, Conditions at the different sites are affected by a number of factors, including grain size, content of organic matter, sediment supply rates, and the ^210^Pb flux^86^. This study reveals that ^210^Pb_ex_ profiles are often shaped by radioactive decay in steady-state sedimentation environments, which are uncommon in the coastal zone due to the variability of sedimentation conditions, population growth, and industrialization. The variations in these patterns are interpreted as changes in sediment accumulation, implying negligible post-depositional mixing^86^.

Similarly, the ^137^Cs activity concentrations varied from 0.20 ± 0.07 to 3.95 ± 0.78, from 0.29 ± 0.04 to 3.25 ± 0.18, from 1.34 ± 0.12 to 5.37 ± 0.43, and from 0.27 ± 0.08 to 2.80 ± 0.41 Bq/kg with an average values 1.22 ± 0.21, 1.79 ± 0.12, 3.36 ± 0.32, and 1.63 ± 0.33 Bq/kg, respectively in cores ED-1, ED-2, ED-3, and ED-4, respectively as shown in (Table 2). Since 1954, ^137^Cs has been released into the environment due to nuclear tests on land, reaching a peak in 1962–1964 when the number of tests increased significantly, causing a spike in ^137^Cs fallout in subsequent years. The use of ^137^Cs as a chronologic indicator relies on identifying its first appearance, which is often obscured by the movement of ^137^Cs across the sedimentary record and its highest contribution to the activity profile^67^. The initial peak of ^137^Cs activation occurred at depths of 42 cm (core ED-1), 48 cm (core ED-2), 36 cm (core ED-3), and 36 cm (core ED-4), which are thought to be related to the Chernobyl disaster. In the same vein, several possibilities could be linked to the second peak of ^137^Cs at depth 12 cm. This peak could be linked to global fallout and the nuclear sector, which could transfer between the Black Sea and the Mediterranean region^87^. It could possibly be attributable to fresh ^137^Cs deposition in the study area after the Fukushima nuclear accident (AD 2011). This is consistent with the distribution of ^137^Cs discovered in the eastern Black Sea coast of Turkey^88^ and the freshwater Hazar Lake in Turkey^89^. Furthermore, the Mediterranean region is already experiencing environmental stress as a result of heat waves, unpredictable precipitation, inadequate water supplies, and drought. As a result, flash floods are rather prevalent in the Mediterranean and are one of the most serious natural dangers in the region^90^. In light of this, we propose that the ^137^Cs peak seen in our sediment cores may be related to the Fukushima disaster, while acknowledging that alternate origins, such as catchment-derived inputs or residual worldwide fallout, cannot be ruled out. The behaviour of ^137^Cs in lake sediment cores is similar to that of ^210^Pb, which could be related to the early events in these cores. ^210^Pb and ^137^Cs may have moved differentially in this soil by means other than physical mixing^91^. ^137^Cs activities in Edku lake (which increased towards the top of the sediment profile (see Fig. 2) also suggests continuous input of ^137^Cs from the catchment through increased soil erosion, since this nuclide is easily transported in dissolved form from upstream soils by surface runoff^10^. Furthermore, The consistent occurrence of elevated Pb-210 and Cs-137 activities especially in the deepest sediment layers can be attributed to the fine-grained nature of pre-AHD deposits, which were primarily composed of silt and clay derived from the Nile’s natural flooding regime. These fine sediments have a high surface area and strong adsorption capacity for radionuclides, enabling them to retain both Pb-210 and Cs-137 more effectively than coarser sediments. Before the construction of the Aswan High Dam, the Nile floodwaters regularly transported fine suspended material rich in organic matter and reactive mineral surfaces, providing ideal conditions for the accumulation of these radionuclides. After the dam’s construction, however, the sediment input to Lake Edku shifted toward coarser, shell-rich materials supplied mainly through agricultural and municipal drains, which reduced the fine fraction available for radionuclide binding.

**Table 2:** The activity concentration (Bq/kg) of ^210^Pb_Tot_, ^226^Ra, ^210^Pb_ex_, ^232^Th and ^40^K in sediment core samples of Edku Lake.

Depth cm	ED-1	ED-2	ED-3	ED-4	Depth cm	ED-1	ED-2	ED-3	ED-4
^210^Pb_Tot_					^226^Ra				
−6.00	81.0 ± 3.26	149.1 ± 12.31	142.3 ± 11.75	64.1 ± 2.87	−6.00	14.0 ± 0.10	98.1 ± 1.78	21.7 ± 0.91	17.6 ± 1.66
−12.00	97.0 ± 4.58	113.4 ± 10.21	167.7 ± 16.59	86.4 ± 3.21	−12.00	14.4 ± 1.47	76.6 ± 1.83	36.2 ± 1.55	17.7 ± 1.84
−18.00	64.0 ± 2.42	69.07± 6.83	98.3 ± 8.84	79.3 ± 3.25	−18.00	13.2 ± 0.74	30.5 ± 0.80	27.1 ± 1.06	19.8 ± 1.30
−24.00	44.8 ± 1.56	44.5 ± 3.68	85.6 ± 7.70	75.8 ± 3.51	−24.00	10.6 ± 0.56	25.5 ± 0.73	21.0 ± 0.75	19.6 ± 1.39
−30.00	24.8 ± 0.97	64.0 ± 6.33	104.1 ± 10.30	47.2 ± 2.23	−30.00	9.48 ± 0.43	24.9 ± 0.61	20.4 ± 1.26	16.5 ± 0.92
−36.00	26.2 ± 0.96	52.4 ± 4.72	85.1 ± 7.66	49.7 ± 2.27	−36.00	10.7 ± 0.45	22.9 ± 0.70	22.2 ± 0.76	17.2 ± 0.93
−42.00	39.8 ± 1.69	47.7 ± 4.29	46.9 ± 4.22	44.6 ± 2.04	−42.00	9.94 ± 0.52	20.9 ± 0.50	22.1 ± 0.74	15.3 ± 0.69
−48.00	17.9 ± 0.71	38.2 ± 3.44	63.6 ± 5.72	38.3 ± 1.83	−48.00	7.44 ± 0.31	21.2 ± 0.55	25.5 ± 0.76	16.9 ± 0.61
−54.00	19.9 ± 0.85	44.4 ± 4.00	40.0 ± 3.60	37.8 ± 1.79	−54.00	7.42 ± 0.34	19.12 ± 0.40	22.9 ± 1.12	16.9 ± 0.64
−60.00	14.0 ± 0.68	28.8 ± 2.59	58.4 ± 5.25	37.5 ± 1.52	−60.00	6.85 ± 0.29	8.17 ± 0.25	16.8 ± 0.51	17.9 ± 1.57
−66.00	18.7 ± 0.68	28.0 ± 2.52	41.3 ± 3.72		−66.00	8.32 ± 0.32	8.60 ± 0.28	18.6 ± 0.59	
−72.00	20.7 ± 0.93	24.7 ± 2.22			−72.00	9.22 ± 0.40	12.43 ± 0.36		
Mean	39.1 ± 1.61	58.68 ± 5.26	84.8 ± 7.8	56.1 ± 2.45	Mean	10.12 ± 0.50	30.74 ± 0.73	23.1 ± 0.9	17.53 ± 1.16
Depth cm	ED-1	ED-2	ED-3	ED-4	Depth cm	ED-1	ED-2	ED-3	ED-4
^210^Pb_ex_					^137^Cs				
−6.00	67.0 ± 3.26	41.3 ± 12.6	120.6 ± 11.79	46.5 ± 3.31	−6.00	2.30 ± 0.54	3.04 ± 0.25	4.41 ± 0.40	1.65 ± 0.40
−12.00	82.6 ± 4.81	36.9 ± 10.4	131.5 ± 16.76	68.7 ± 3.70	−12.00	3.95 ± 0.78	2.84 ± 0.21	5.37 ± 0.43	2.13 ± 045
−18.00	50.8 ± 2.53	38.6 ± 6.88	71.2 ± 8.71	59.5 ± 3.50	−18.00	2.77 ± 0.29	3.25 ± 0.18	3.88 ± 0.32	2.80 ± 0.41
−24.00	34.2 ± 1.66	19.0 ± 3.75	64.6 ± 7.74	56.2 ± 3.77	−24.00	1.24 ± 0.18	2.27 ± 0.16	3.04 ± 0.25	1.84 ± 0.32
−30.00	15.3 ± 1.06	39.0 ± 6.36	83.7 ± 10.37	30.7 ± 2.41	−30.00	0.46 ± 0.09	2.21 ± 0.12	3.69 ± 0.42	1.26 ± 0.29
−36.00	15.5 ± 1.06	29.6 ± 4.77	62.9 ± 7.70	32.5 ± 2.45	−36.00	0.47 ± 0.11	2.12 ± 0.15	4.23 ± 0.74	1.42 ± 0.23
−42.00	29.8 ± 1.77	26.8 ± 4.32	24.8 ± 4.29	29.3 ± 2.16	−42.00	1.20 ± 0.22	1.15 ± 0.08	2.59 ± 0.20	1.17 ± 0.21
−48.00	10.5 ± 0.78	17.0 ± 3.48	38.1 ± 5.78	21.4 ± 1.93	−48.00	0.58 ± 0.09	2.34 ± 0.13	3.69 ± 0.21	0.92 ± 0.16
54.00	12.5 ± 0.91	25.3 ± 4.02	17.0 ± 3.77	20.9 ± 1.91	54.00	0.72 ± 0.09	1.27 ± 0.07	2.94 ± 0.28	0.44 ± 0.13
−60.00	7.11 ± 0.74	20.6 ± 2.60	41.5 ± 5.28	19.6 ± 2.18	−60.00	0.28 ± 0.06	0.35 ± 0.04	2.58 ± 0.14	0.27 ± 0.08
−66.00	10.3 ± 0.75	19.4 ± 2.53	22.7 ± 3.76		−66.00	0.42 ± 0.06	0.29 ± 0.04	1.34 ± 0.12	
−72.00	11.5 ± 1.02	12.2 ± 2.25			−72.00	0.20 ± 0.07	0.38 ± 0.04		
Mean	32.5 ± 1.66	27.9 ± 5.33	61.7 ± 7.8	38.5 ± 2.73	Mean	1.22 ± 0.21	1.79 ± 0.12	3.36 ± 0.30	1.63 ± 0.33

### Numeric ^210^Pb_ex_ and ^137^Cs dating models

**Fig. 3:**
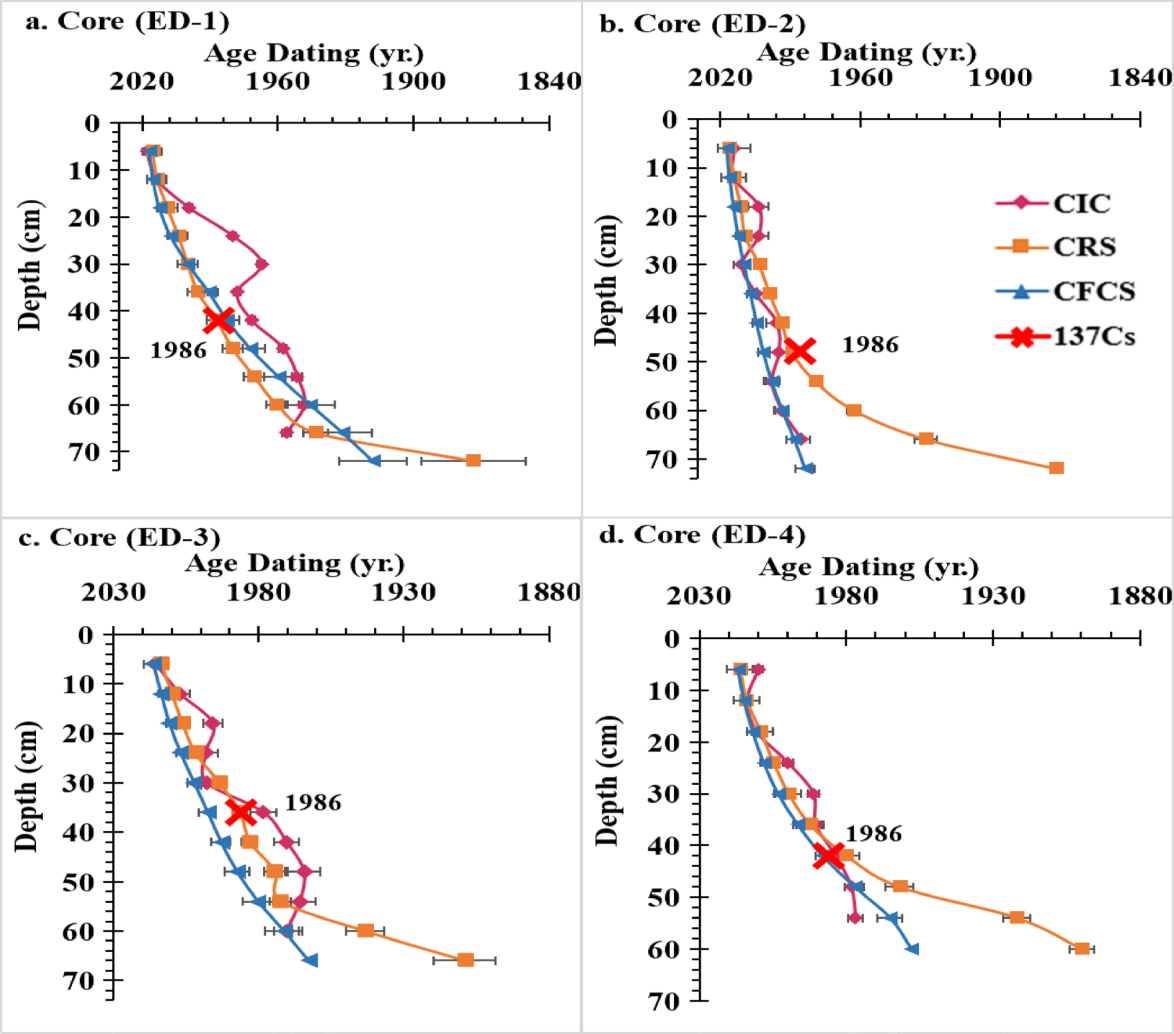
Comparison of the depth-age relation derived from CIC, CRS, CF-CS models and the ^137^Cs markers in all sediment core samples of Edku Lake.

The tracer isotopes that are frequently used in lake sediment dating are ^210^Pb_ex_ and ^137^Cs. A ^210^Pb_ex_ activity profile can be interpreted using a variety of models; the projected dates are determined by whatever model best captures the ^210^Pb geochemistry^83^. Table 3 and Fig. 3 show that sediment ages were established using CIC, CRS, and CFCS models and validated using the ^137^Cs time marker on four Edku Lake cores. Similar to previous studies, chronologies generated by different models showed significant differences in the same core^10,13^. Furthermore, in certain cores, age estimation using the CIC model resulted in a notable time inversion. The CIC model predicted an earlier date for a deeper layer with higher activity due to the significant non-monotonic change in ^210^Pb_ex_ activity, which is physically inconceivable^28^. The chronology of the CF-CS and CRS models revealed an appropriate correspondence in the upper strata of the core ED-1. However, the ages calculated by the CIC model differ significantly from those estimated by other models. The differences in the dates of the CIC model revealed that the initial ^210^Pb concentrations varied for various reasons. Because the ^210^Pb profile of this lake is not monotonic, the CIC model generated significant temporal inversions across all profiles, which is consistent with previous research^91,92^. The age estimated by CIC, CF-CS and CRS represented a good fit at all depth of the cores ED–2 and ED–4 but at the deepest layer CRS gave older age than CIC and CF-CS (see Fig. 3). In the core ED–3, there was a good agreement in the ages estimated by CIC and CRS models upper depth 42, below that depth CIC model gave younger age than CRS model. Typically, the CRS model produced older ages than other models. For all studied cores, the CIC and CF-CS models produced younger ages for the underlying layers, while the CRS model provided younger ages for core ED-1 than the CF-CS model (see Fig. 3).

Based on our findings, the CRS model had the best fit with the ^137^Cs chronometer. The rapid development of urbanization in these lakes during the last half of the century may have influenced the distribution of ^210^Pb_ex_ in sediments, leading to overestimation or underestimation of chronologies by CIC and CFCS models^25^. This reprocessed ^210^Pb_ex_ fallout, primarily due to sediment erosion, is anticipated to account for a significant amount of the initial ^210^Pb_ex_ inventory in the research locations, as demonstrated in other investigations^29,93^. The CIC and CF-CS models were unable to produce a trustworthy chronology because they are significantly influenced by the initial ^210^Pb concentrations. However, changes in initial concentrations and sedimentation rates at different strata have no effect on the CRS model^91^. Because Lake Edku receives precipitation and industrial and agricultural runoff as its main sources, the CRS model appears to be more appropriate for the lake. Furthermore, because of the uneven water discharge, the sedimentation rate may not have been steady.

**Table 3:** Calendar dates (yrs.) estimated by CIC, CRS, and CF-CS ^210^Pb dating models in Edku lake sediment cores.

Depth	Core ID	CIC	CF-CS	CRS	Core ID	CIC	CF-CS	CRS
−6.00	ED-1	2018 ± 1	2017 ± 1	2016 ± 4	ED-2	2014 ± 7	2017 ± 1	2016 ± 1
−12.00		2013 ± 1	2015 ± 1	2013 ± 4		2014 ± 5	2016 ± 1	2014 ± 1
−18.00		1999 ± 1	2012 ± 1	2009 ± 4		2004 ± 4	2014 ± 1	2011 ± 1
−24.00		1980 ± 1	2008 ± 2	2004 ± 4		2004 ± 4	2013 ± 1	2009 ± 1
−30.00		1967 ± 2	2000 ± 3	2000 ± 4		2011 ± 4	2010 ± 1	2003 ± 1
−36.00		1978 ± 2	1991 ± 4	1995 ± 4		2005 ± 4	2007 ± 1	1999 ± 2
−42.00		1972 ± 2	1982 ± 5	1987 ± 4		1996 ± 4	2005 ± 2	1994 ± 2
−48.00		1958 ± 2	1972 ± 7	1980 ± 4		1995 ± 4	2002 ± 2	1989 ± 2
−54.00		1952 ± 2	1960 ± 9	1971 ± 5		1998 ± 3	1998 ± 2	1979 ± 2
−60.00		1949 ± 2	1946 ± 11	1960 ± 5		1994 ± 3	1993 ± 3	1963 ± 3
−66.00		1956 ± 2	1931 ± 13	1943 ± 6		1985 ± 4	1988 ± 4	1932 ± 5
−72.00			1918 ± 15	1874 ± 23			1984 ± 4	1876 ± 2
−6.00	ED-3	2016 ± 3	2016 ± 1	2014 ± 1	ED-4	2010 ± 2	2017 ± 1	2016 ± 4
−12.00		2007 ± 3	2014 ± 1	2010 ± 1		2014 ± 2	2015 ± 0.3	2014 ± 4
−18.00		1996 ± 3	2011 ± 2	2006 ± 1		2011 ± 2	2012 ± 0.5	2009 ± 4
−24.00		1998 ± 3	2007 ± 2	2002 ± 1		2000 ± 2	2008 ± 1	2005 ± 4
−30.00		1998 ± 3	2002 ± 3	1994 ± 1		1991 ± 2	2003 ± 1	2000 ± 4
−36.00		1978 ± 3	1998 ± 4	1987 ± 2		1990 ± 2	1997 ± 2	1992 ± 4
−42.00		1970 ± 4	1993 ± 5	1983 ± 2		1984 ± 2	1988 ± 2	1980 ± 5
−48.00		1964 ± 4	1988 ± 7	1974 ± 2		1978 ± 2.09	1977 ± 3	1962 ± 5
−54.00		1966 ± 4	1980 ± 8	1963 ± 3		1977 ± 2	1965 ± 4	1922 ± 5
−60.00		1970 ± 3	1971 ± 10	1943 ± 4			1958 ± 5	1900 ± 4
−66.00			1963 ± 12	1909 ± 1				

**Fig. 4:**
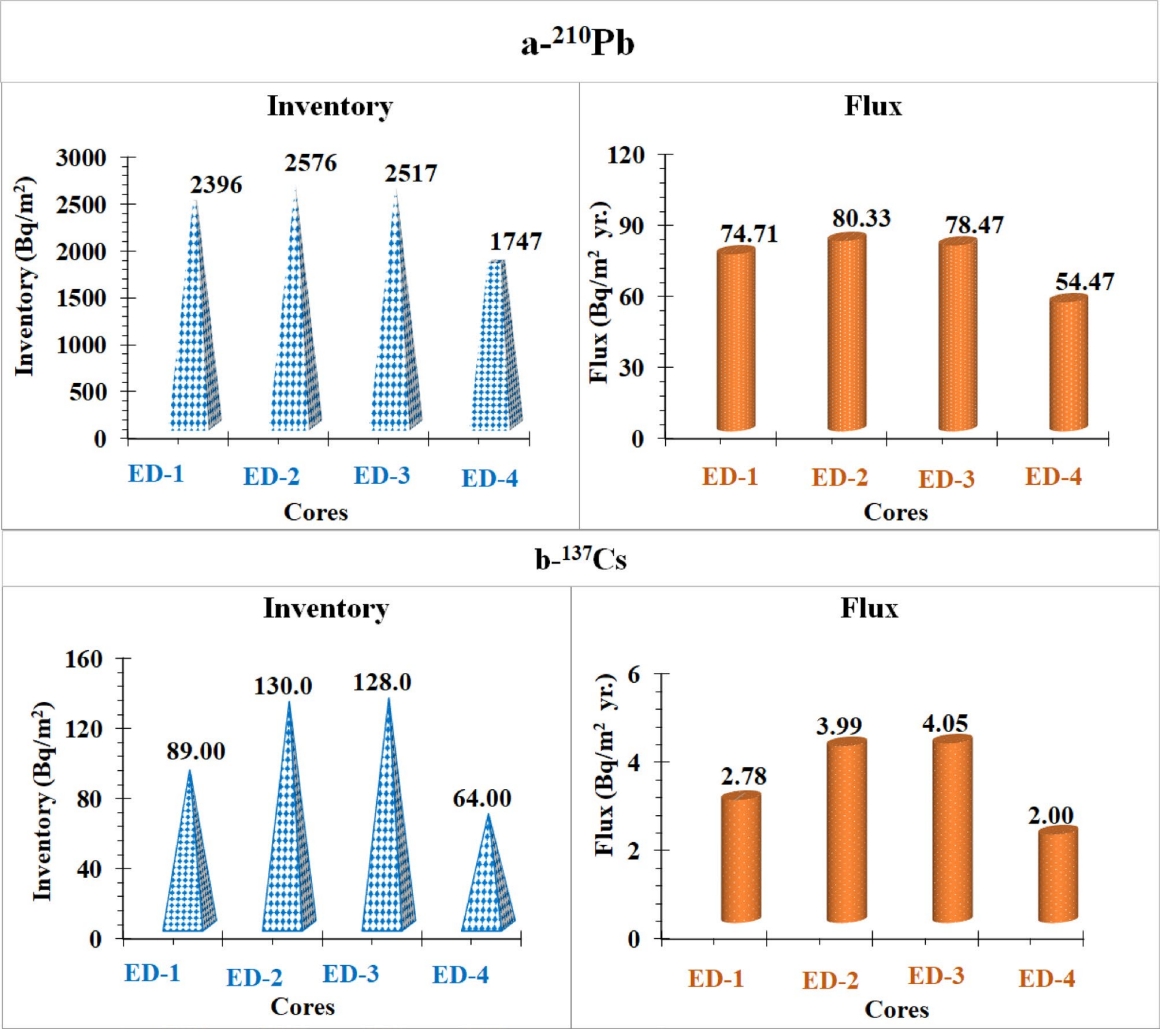
Radionuclide inventories and Atmospheric fluxes of ^210^Pb_ex_ and ^137^Cs in sediment core samples of Edku Lake.

The CRS model assumes that the excess ^210^Pb input to sediment at a specific location remains generally stable across time. Given that the CRS model revealed significant variability among the four cores, a total inventory of ^210^Pb is required to estimate the chronology. Fig. 4a displayed the total inventories and associated flux of ^210^Pb_ex_. The examined cores were ordered in the following order by the total ^210^Pb_ex_ inventories: ED–2 > ED–3 > ED–1 > ED–4. However, ^210^Pb_ex_ fluxes in all Edku lake cores (mean flux 72.00 Bq/m² yr.) were within the normal range of ^210^Pb_ex_ water-sediment deposition flux in northern coastal zones (100–450 Bq/m² yr.) over the latitudinal band 10–30°N^86^. Elevated ^210^Pb_ex_ fluxes were attributed to erosion, mostly through rivers, and a concentration of fine sediment in sample sites. The atmospheric flux of ^210^Pb_ex_ can vary with latitude due to a variety of factors such as rainfall regime and geographical location^91,94^. The average values of ^210^Pb fluxes in Edku Lake exceeded the annual average ^210^Pb atmospheric flux in Lake Qarun (102.7 ± 5.2 Bq/m²/yr)^91^. The atmospheric flux of ^210^Pb_ex_ might vary depending on latitude and other factors such as the weather pattern and geographical location^91,94^. The average value of the ^210^Pb fluxes in Edku Lake was significantly higher than the average annual ^210^Pb atmospheric flux in Lake Qarun (102.7 ± 5.2 Bq/m²/yr)^91^. In Fig. 4-b, the total inventory of ^137^Cs in sediment cores is shown. The ^137^Cs inventory was lower than the benchmark values (470 Bq/m²) ^15^, which could be attributed to ^137^Cs being removed from lake outflows.

The ^137^Cs peak can be utilized as an independent stratigraphic chronomarker. ^137^Cs activity in all analyzed cores showed two distinct peaks. In the cores ED-1 and ED-4 profiles, among the CIC, CRS, and CF-CS models, the CF-CS plot appears to correlate well with the ^137^Cs technique-based chronology (see Fig. 3). The CRS model chronology was much closer and more compatible with the ^137^Cs peak marker found in all Edku Lake cores analyzed. The highest part of the ^137^Cs levels in the 18-centimeter layer of all the studied sediment cores might be due to worldwide pollution and nuclear energy. This pollution might be found between the Black Sea and the Mediterranean region^87^. A new ^137^Cs deposition may be associated with it in our study following the nuclear disaster in Fukushima (AD 2011). This leads to the discovery of ^137^Cs on the eastern coast of the Black Sea in Turkey^88^ and in the southern Hazar Lake in Turkey^89^. The Mediterranean region is also facing environmental challenges, including heatwaves, unpredictable precipitation, water scarcity, and droughts. Flash floods are a frequent occurrence in the Mediterranean, playing a significant role in the region’s most devastating natural disasters^90^. According to the CRS model, the impact of Nile floodwaters before the construction of the Aswan High Dam in the 1960 s was more pronounced at sites ED-3 and ED-4 (54 cm at ED-3 and 48 cm at ED-4) compared to ED-1 and E2 (60 cm). This variation indicates stronger erosion of sediment layers caused by the force of floodwater flow. Evidence of this can be seen in the sediment characteristics of Edku Lagoon, where sediment texture analyses reveal a distinct stratification pattern: a surface layer of dark gray silty clay with few shell fragments, a middle layer of silty sand rich in shell fragments, and a basal layer of grayish silty clay devoid of shells^40^. Furthermore, dating of sediment layers from four cores deposited after the 1960 s (following the construction of the Aswan High Dam) shows significant age variations, reflecting the varying influences of discharges from agricultural and municipal sources. After the dam’s completion, the lake’s primary inflows shifted from Nile floodwaters to agricultural and municipal drains, mainly from Kom Belag and Bersik, leading to noticeable changes in sediment deposition patterns.

### Sedimentation rates

**Fig. 5:**
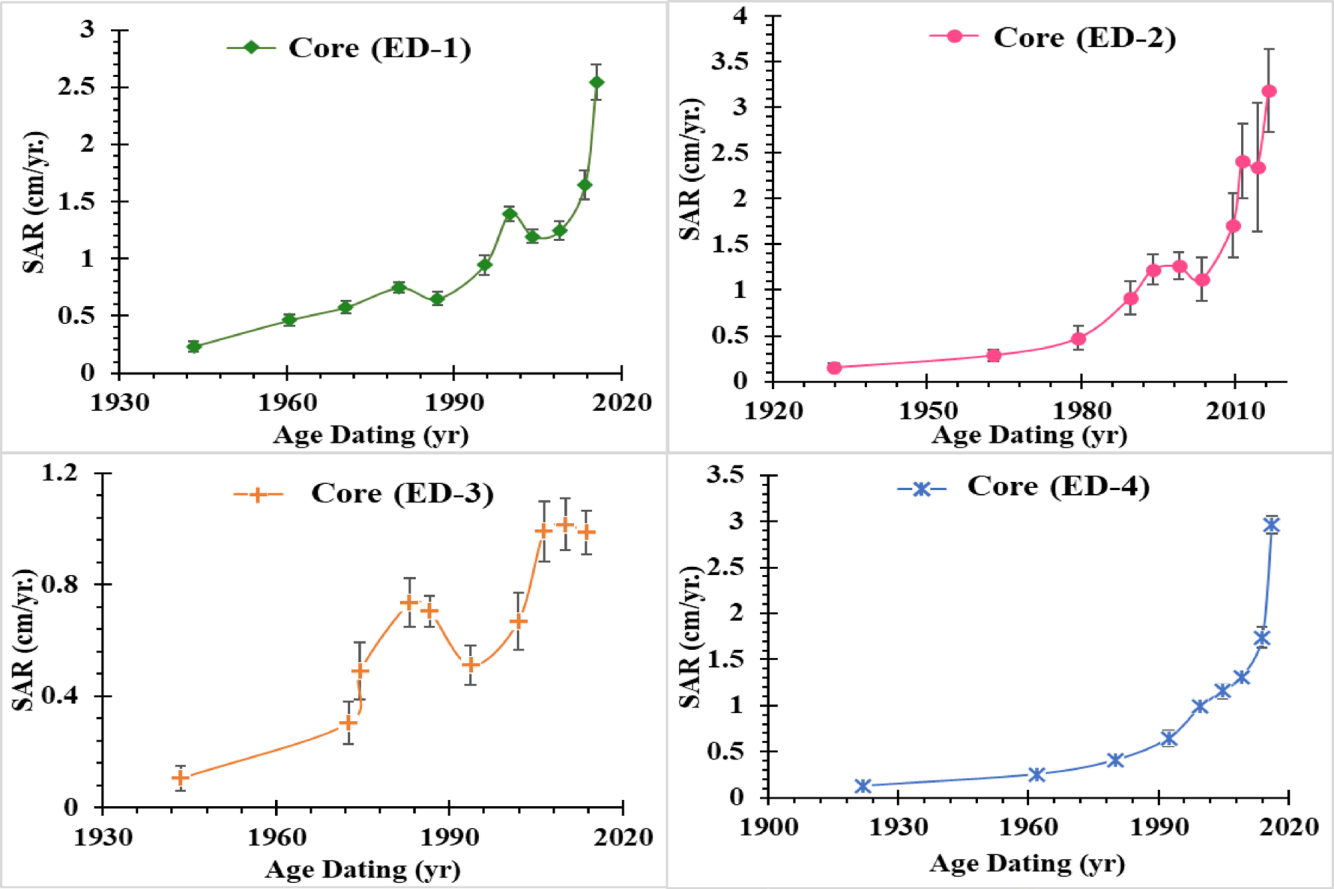
Historical changes in sediment accumulation rates (SAR) derived from CRS model in the selected cores of Edku Lake.

This study used radionuclide tracers (^210^Pb and ^137^Cs) to determine precise sedimentation rates in vulnerable Edku Lake and develop more targeted restoration strategies. Different sedimentation rates were obtained due to discrepancies between several models. Utilizing the CRS model, the vertical depth profile of core sediments in Lake Edku from 1900 to 2018 was ascertained. Sedimentation rates were estimated at each core location using the CFCS and CRS models (SAR, cm/yr.; MAR, gm/cm^2^yr.) (see Fig. 5 and Table S1). The CF-CS model exhibited SAR and MAR values of 0.80 ± 0.12 cm/yr. and 0.12 ± 0.02 gm/cm^2^yr. (core ED–1), 1.65 ± 0.30 cm/yr. and 0.25 ± 0.04 gm/cm^2^yr. (core ED–2), 1.20 ± 0.02 cm/yr. and 0.09 ± 0.01 gm/cm^2^yr.  (core ED–3), and 0.96 ± 0.08 cm/yr. and 0.09 ± 0.01 gm/cm^2^ yr. (core ED–4), respectively. For the CRS model, the MAR values ranged from 0.06 ± 0.01 to 0.27 ± 0.02 gm/cm^2^yr., from 0.03 ± 0.01 to 0.19 ± 0.04 gm/cm^2^yr., from 0.02 ± 0.00 to 0.07 ± 0.01 gm/cm^2^yr., from 0.01 ± 0.00 to 0.09 ± 0.01 gm/cm^2^yr., with an average 0.14 ± 0.01, 0.14 ± 0.02, 0.05 ± 0.01, and 0.07 ± 0.00 gm/cm^2^yr. in cores ED–1, ED–2, ED–3, and ED–4, respectively. otherwise, the SAR values of CRS model varied from 0.21 ± 0.02 to 4.45 ± 0.15 cm/yr., from 0.14 ± 0.04 to 6.38 ± 0.37 cm/yr., from 0.10 ± 0.02 to 1.63 ± 0.11 cm/yr., and from 0.07 ± 0.01 to 4.42 ± 0.16 cm/yr., with an average value of 1.32 ± 0.0 cm/y, 1.67 ± 0.20 cm/yr., 0.71 ± 0.09 cm/yr., and 1.11 ± 0.07 cm/yr. in cores ED–1, ED–2, ED–3, and ED–4, respectively. The sedimentation rates showed a sharp increase from the late 1980 s and early 2000 s until reaching the maximum value in 2018, (4.45 ± 0.15, 6.38 ± 0.37, 1.63 ± 0.11, and 4.42 ± 0.16 cm/yr. in core ED–1, ED–2, ED–3, and ED–4, respectively) as shown (Fig. 5). The average sedimentation rates arranged the examined cores in the following order: ED-2 > ED-1 > ED-4 > ED-3, with an increase towards the drains. The average values of sedimentation rates (SAR cm/yr.) estimated by model CRS was slightly agree with the CF-CS model and ^137^Cs- time marker estimations in all the studied cores except ED–4 (see Table 1S).

The historical trends in sedimentation rates of Lake Edku reveal the influence of several key environmental and anthropogenic events, which can be divided into three distinct periods. The first period (1960–1970) corresponds to the construction of the Aswan High Dam (AHD), which interrupted the Nile’s natural sediment supply delivered through its annual floods. The second period, post-1980, is characterized by extensive land reclamation, agricultural expansion, and the growth of industrial activities around the lake. The third period, representing the Modern Era (2000–2010 s), is marked by the rapid increase in fish farming and a significant reduction in the lake’s surface area. Sedimentation records show that prior to the 1960 s, the sedimentation rate in the four cores studied was primarily controlled by the Nile’s natural flooding regime. However, after the construction of the AHD, a substantial shift in the lake’s water sources occurred. The dam’s obstruction of the Nile’s flow transformed Lake Edku’s main water and sediment inputs into highly polluted agricultural, industrial, and municipal drainage water discharged from major drains such as Kom Belag and Bersik^59,60^. This shift is reflected in the observed sedimentation rates, which doubled compared to the pre-1960 levels.

In the Modern Era, sedimentation rates continued to rise, likely due to the increased pollutant and sediment loads, as well as the proliferation of fish farms surrounding the lake. During this period, Lake Edku entered a state of hyper-eutrophication and severe pollution stress. Studies from the 2000 s and 2010 s consistently classify the lake as a shallow, hyper-eutrophic basin receiving excessive nutrient and contaminant inflows^51^. Research from 2012 to 2013 further confirmed that extremely high nutrient concentrations in the drainage water were the main cause of the lake’s degraded ecological condition^51^. Furthermore, intensive land reclamation and land-use changes in recent decades have drastically reduced the lake’s open-water area^60,63^. This reduction has concentrated both pollutants and sediments within a smaller volume of water, significantly accelerating sediment accretion and infilling rates. As a result, the detrimental effects of drainage inflows have become increasingly pronounced and ecologically damaging.

Table 4 shows that the estimated average sedimentation rates for the study lagoon exceed those for other coastal habitats. The main factor contributing to Edku Lake’s increasing sedimentation rates may be linked to the lake’s area shrinking since 1964 as a result of large areas of the eastern shore being taken for agricultural and urban purposes^95^. Furthermore, Edku Lake receives a significant amount of drainage water from domestic, agricultural, and industrial urban effluents, as well as fish farms^65^.

**Table 4:** Sediment accumulation rates (SAR cm/yr.) in coastal environments worldwide.

Sites	SAR (cm/yr.)	Reference
Edku Lake, Egypt	0.71–1.67	Present study, 2025
Burullus Lake, Egypt	0.86–1.08	^96^
Lake Qarun, Western Desert, Egypt	0.45 ± 0.10	^91^
Burullus lagoon, Egypt	0.70	^97^
Edku lagoon, Egypt	0.21–0.33	^15^
San Jose Lagoon, Mexico	0.16–0.51	^98^
Kodaikanal Lake, South India	0.51 ± 0.14	^99^
Anzali Lagoon, Caspian Sea	0.76 ± 0.12	^7^
Lake Pykara, Tamil Nadu, India	0.70 ± 0.07	^8^
Satopanth Tal Garhwal Himalaya	0.79 ± 0.04	^100^
Northern Beibu Gulf, South China sea	0.79	^10^

### Historical profiles of major and trace elements concentrations in the sediment cores

**Fig. 6:**
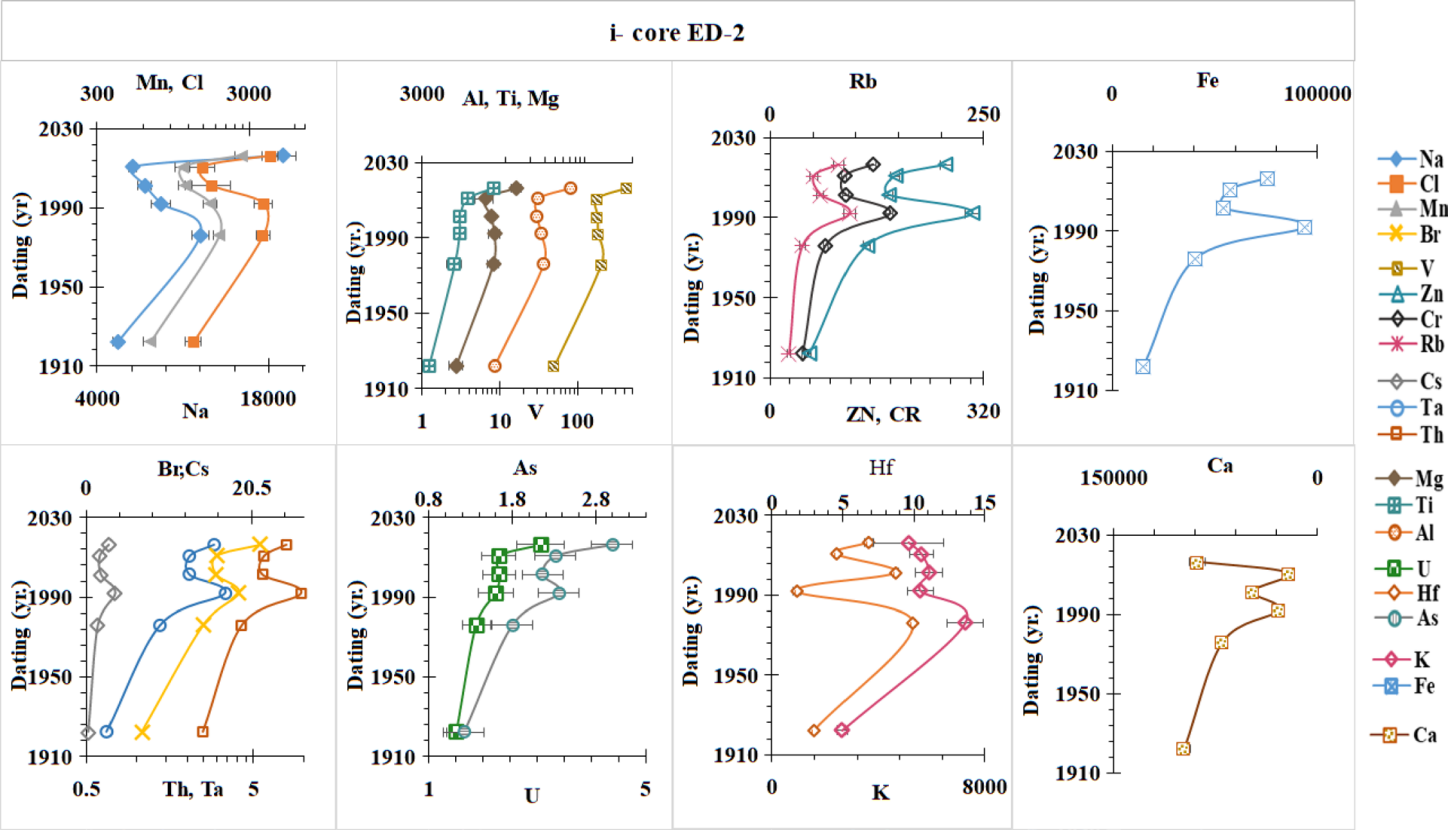

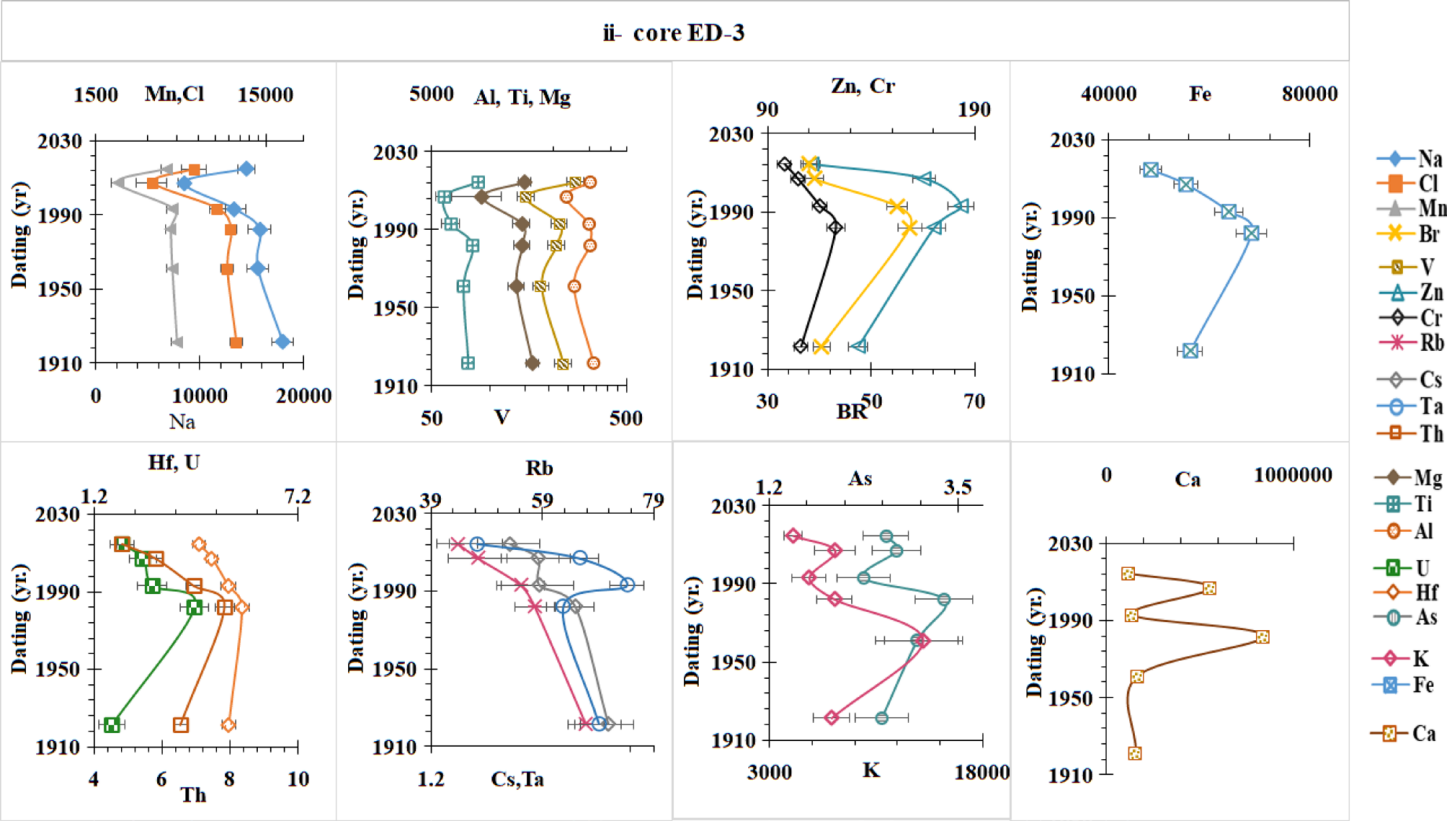
Vertical distribution of major and trace elements concentrations in the sediment cores of Edku Lake.

The element concentration profiles in dated sediment cores (ED–2 and ED–3) of Lake Edku were determined to obtain information on the flux of different elements and their anthropogenic contribution. The concentration of the elements was measured by INAA (µg/g) in the sediment cores of the two lakes (Table S2 and Fig. 6). The metal concentrations obtained were compared with the corresponding values from the literature of^73^ for continental shale abundance. The concentrations of Br, Fe, Mg, V, Cl, Mn, Cr, Ca, Mn, Zn, Ti and Na are significantly higher than the corresponding values in continental shale deposits^73^. For the other metals, such as Al, K, Sc, As, Rb, Cs, Th, U, Ta and Hf, the values are below or slightly above the background values in continental shale deposits. According to these results, there is a significant presence of polluting elements in selected core sediments of two lakes associated with the discharge of untreated wastewater. Lake Edku receives a total of 592 × 10^6^ m^3^/yr. and 348 × 10^6^ of untreated drainage water (domestic, agricultural and industrial) per year from the El-Khairy and Barsik drains, respectively^65^. Mg, Br, Na, and Cl concentrations were found to be greater in the lake’s northern parts and nearby El-Boughaz due to infiltration of seawater^101,102^. Furthermore, large quantities of (magnesium) Mg and (sodium) Na were found near the outflows, which could have been caused by agricultural waste^101^. High levels of bromine (Br) can also be attributed to its use in agricultural fumigants and as a component of K fertilizers. Calcium (Ca) concentrations in sediments were approximately seven to eight times higher in comparison to the presence of continental shale. This might potentially be due to large accumulations of shell debris in the sediments. In addition, the calcium content of the lake sediments was best explained by an increase in both the proportion of agriculture in the lake catchments and the average soil pH in the catchment^103^.

The chronology of the selected cores was estimated in the section "Numeric ^210^Pb_ex_ and ^137^Cs dating models". The changes that have been undergone by Edku Lake over the past 100 years in terms of human activity and the aquatic environment are to be determined. In core ED-2 (Fig. 6-i), the concentrations of Mn, Cl, Na, Fe, Zn, Cr, Rb, Br, Cs, Th, and Ta increased from 1922 to 1992, decreased from 1992 to 2011, and then increased again from 2011 to 2016. These elements reached their maximum values in 1992 and 2016. The maximum values of Al, Ti, Mg, V, As, and U were found in 2016, with no variation in concentrations from 2011 to 1976, followed by a sharp decrease from 1976 to 1922. K and Hf showed similar vertical trends, with maximum values found in 1976 and 2001. The Ca concentration displayed higher values in the bottom layers. In core ED-3 **(**Fig. 6-ii**)**, the concentrations of Na, Cl, Mn, Al, Ti, Mg, and V varied minimally from 2016 to 1921, with higher values in the bottom layers. Concentrations of Br, Cr, Zn, Fe, Hf, U, and Th increased to maximum values in 1982, then decreased from 1982 to 1921.

The sediments carried by the Nile’s annual floods were naturally rich in structural elements such as silicon (Si), iron (Fe), aluminum (Al), magnesium (Mg), calcium (Ca), and potassium (K), along with other essential trace elements including manganese (Mn), zinc (Zn), and phosphorus (P)^62,104^. However, following the 1960 s, the construction of the Aswan High Dam (AHD) blocked the Nile’s natural sediment supply, interrupting the influx of these nutrient-rich materials, as observed in Edku Lake. The sediment of lake after 1970, slight decreases in the concentrations of Al and Mg were observed, confirming their origin from Nile-derived sediments. In contrast, Ca and Fe, which were naturally abundant in sediments before 1970, showed a marked increase after the 1990 s, primarily due to intensified human activities such as industrial development and agricultural expansion. Similarly, Mn concentrations reached their natural maximum during the 1970 s under the influence of Nile flood inputs, followed by a sharp decline after the construction of the AHD. Later, in the 2000 s, Mn levels rose again, coinciding with extensive land reclamation and increased agricultural activity and fish farms around the lake. Furthermore, potassium (K) concentrations reached their maximum levels around 1970, reflecting its natural abundance in Nile flood sediments, where it commonly occurs as a constituent of K-feldspars and mica minerals. However, following the construction of the Aswan High Dam, potassium levels declined sharply due to the interruption of the Nile’s natural sediment supply^100^. Clearly, most element concentrations in the investigated cores remained relatively constant before the 1990s. This demonstrates that these elements were largely impacted by natural sources, such as the weathering of rocks in basin^105^ and the Nile’s natural sediment supply rather than by human activity. Consequently, urbanization and industrialization over the past few decades have led to an increase in pollutants from a variety of sources, including wastewater, industry, and agriculture^106^. Additionally, between 2000 and 2018, some areas of the lake were reclaimed for agricultural purposes, resulting in notable changes in the area surrounding Edku Lake^50^.

Edku Lake’s metal concentrations are contrasted with those of other coastal regions in Table 5. In comparison to other maritime areas, Edku Lake has a lower concentration of arsenic (As). On the other hand, the sediments of Edku have higher levels of Mn, Cr, Zn, Ti, Fe, V, and Ca than the sediments of the previous marine ecosystems, which suggests that the discharge of waste products from human activity has landed in industrial, domestic, and agricultural activities. However, the comparison of element concentrations in sediments worldwide may entail limitations due to the diversity of ecosystems and the types of human activities that impact them as well as sediment characteristics, such as mineralogical composition, grain size distribution, and organic matter and carbonate content^107^.

**Table 5:** Comparison of the metal concentrations (µg/g) in the sediment cores of Edku lakes with different countries.

Locations		Al	Ca	Ti	Cr	V	Mn	Fe	Zn	As	Reference
Edku Lake	ED-2	86,727	59,151	10,009	115	200	1522	56,221	189	2.14	Present Study
	ED-3	90,276	329,502	9295.33	109	215	3819	58,395	153	2.77	
	Avg	88501.5	194,326	9652	112	207	2670	57,308	171	2.45	
Burullus Lake	BU-2	89,919	170,962	9328	158.02	250	1097	68,839	230.46	2.66	^32^
	BU-3	59,507	71,069	7426	145.11	149	870.83	54,316	174.15	3.75	
	BU-3	78,373	138,739	11,189	125.65	205	1146	52,425	180.78	1.93	
	Avg	75,933	12,692	9314	143	201	1038	58,527	195.13	2.78	
Sundarban mangrove sediments forest of Bangladesh (C1)		88,400	18,800	⸺	⸺	87	⸺	32,700	49.1	4.8	^22^
Vietnam’s Mong Cai area		⸺	⸺	⸺	20.60	30.73	118.52	25,400	41.35	12.49	^69^
Sundarbans mangrove forest, Bangladesh (Core 1)		⸺	⸺	⸺	81.3	84	696	42,800	⸺	81.8	^108^
Southwest China, Erhai Lake		80,800	⸺	7700	159	⸺	⸺	64,600	146	⸺	^109^
Marine sediments from Ría de Muros (NW Iberian Peninsula) M12GC		67,500	72,000	3700	⸺	98.4	233	27,000	79.2	16.5	^26^
Western Turkey, Lake Karagöl (S 1)		18,400	⸺	⸺	36.9	⸺	331	⸺	42.4	3.6	^110^
Lake Chenghai, China (CH3)		100,235	19997.9	6204.5	100.2	172.9	942.1	48491.5	128.7	⸺	^111^
Weihe River		⸺	⸺	⸺	52.85	⸺	603.99	⸺	66.19	11.54	^112^

### The statistical analysis

#### The coefficient of variation (CV)

**Fig. 7:**
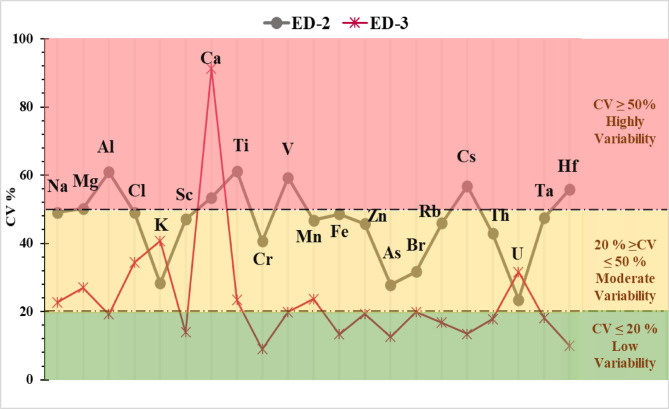
Coefficient of Variation (CV%) of all selected elements in the cores of Edku Lake.

The coefficient of variation (CV) is used to examine the variability in the distribution of elements^113^. Relatively low CV values are found in elements dominated by natural sources, but CV values are significantly higher in elements influenced by man-made sources^80^. Variability is categorized as follows: CV < 20% indicates low variability, 20% ≤ CV ≤ 50% indicates moderate variability, CV > 50% indicates high variability, and CV > 100% indicates extremely high variability. As shown in Table S2 and Fig. 7, the coefficient of variation (CV%) was calculated for all elements in cores ED-2 and ED-3. The high variability of the elements analyzed in the ED-2 core may be due to the large amount of runoff from domestic, agricultural, industrial, and urban sources, as well as runoff from fish farms. Additionally, drainage water moves through the lake from the west and south towards the north and eventually into the sea^67^. The significant increase in Ca in ED-3 is due to several factors. First, remarkable agricultural development and rapid climate warming exacerbated chemical weathering and Ca migration. Second, the calcium in the lake water was more susceptible to evaporation due to the warmer, drier climate. Additionally, the lake’s productivity increased due to the deposition of more reactive nitrogen, which consumed more CO₂ and HCO₃⁻ in the lake water. This increased the lake water’s pH and deposited more Ca^114^. The high variability of V in some cores can be attributed to industries such as oil, petroleum, and steel^115^.

#### Principal component analysis (PCA)

**Fig. 8:**
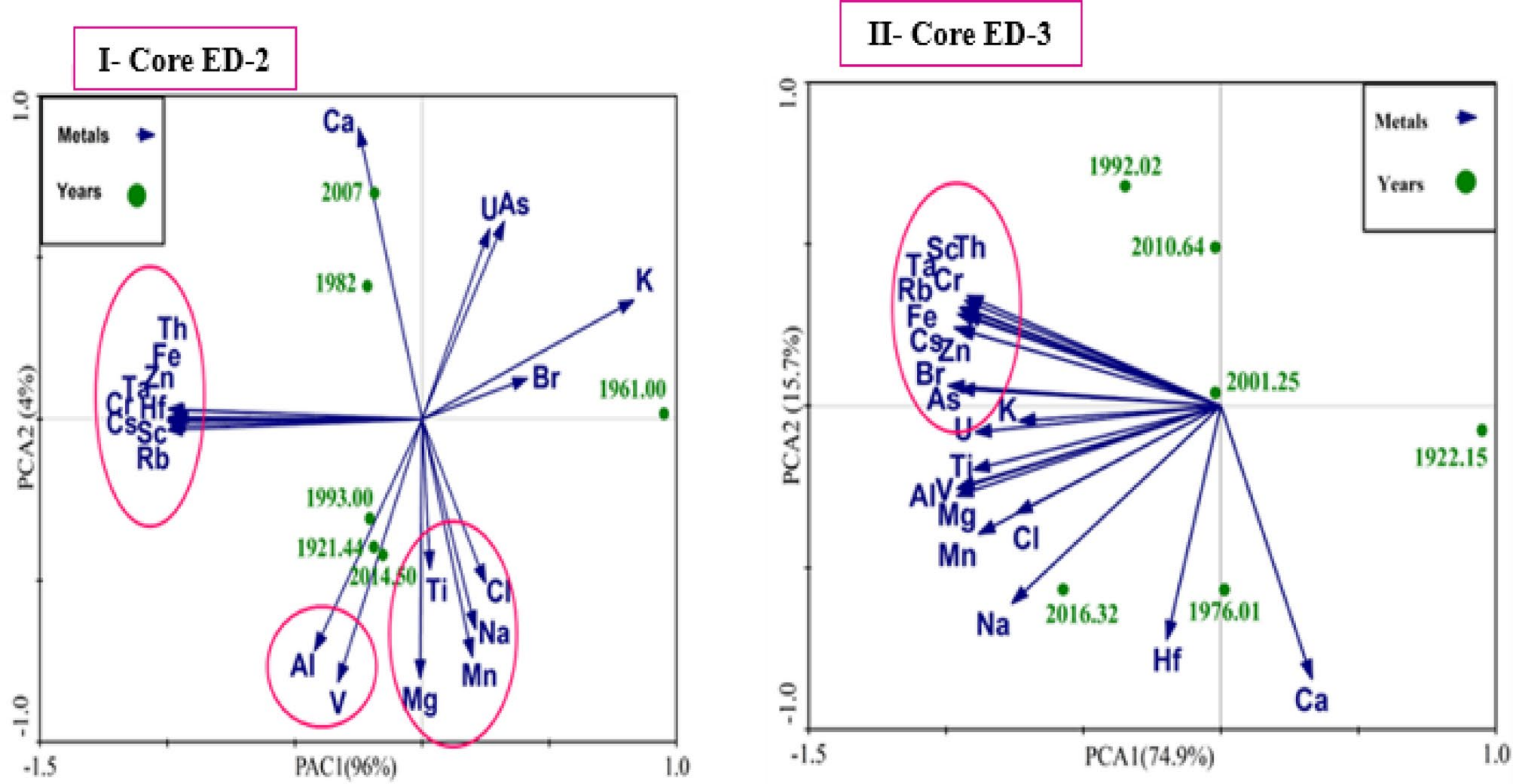
Principle component analysis (PCA) of studied elements with age dating of sediment in cores of Edku Lake.

A principal components analysis (PCA) was carried out using Canoco 5 software on two sediment cores from Edku Lake. The PCA was also used to group the heavy metals and infer their hypothetical source. Element concentration data from Edku core sediments indicate that not all examined heavy metals have common origins and that their behavior during deposition can be unpredictable. Therefore, variations in element content in sediments are closely related to terrestrial inputs, sea-level changes, shifts in provenance, and dynamic depositional environments^71^. The PCA analysis classified metal sources into three major categories, as illustrated in Fig. 8. As a result, these metals primarily originate from the same sources of pollution and natural weathering^116^, as evidenced by their relationship with clay minerals^117^. It has been established that elements like manganese (Mn), sodium (Na), chlorine (Cl), and bromine (Br) may serve as indicators of the salinity of marine water. Elements such as Potassium (K), Iorn (Fe), Thorium (Th), Rubidium (Rb), Scandium (Sc), Titanium (Ti), Magnesium (Mg), Chromium (Cr), and Aluminum (Al) can suggest a terrigenous origin^98^. In contrast, elements like Hf, As, Cr, V, and Zn may directly result from the release of waste materials from industrial, aquatic, and urban areas that have metal pollution^118^.

### Reconstruction of historical evolution based on enrichment factor (EF) and depositional metal flux

#### Enrichment factor (EF)

**Fig. 9:**
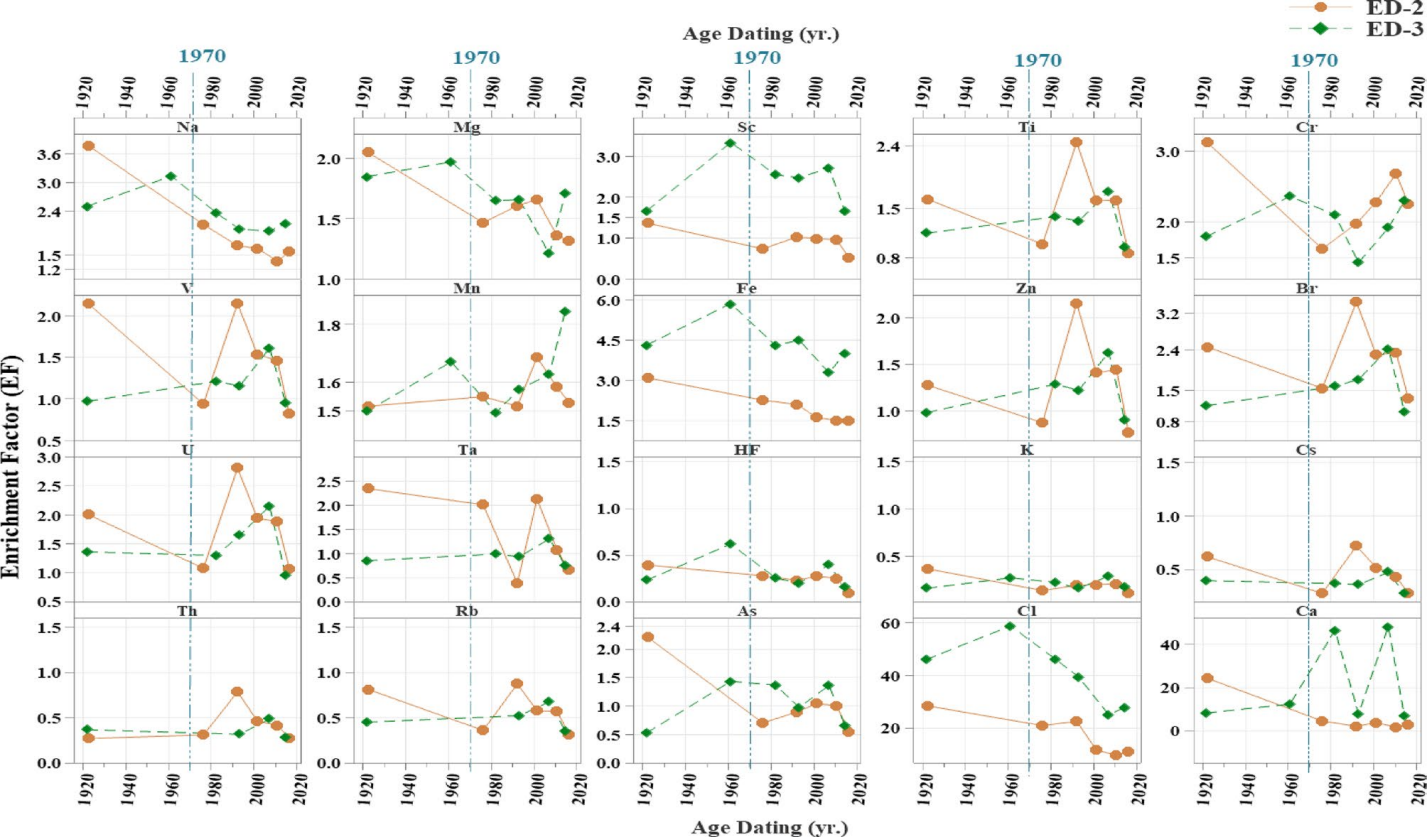
Historical variations of Enrichment Factors (EF) in the studied cores of Edku Lake.

The enrichment factor (EF) has been used to estimate metal contamination over the past decades. The results are presented in Table 6 and Fig. 9. The high EF values indicate that anthropogenic pollution needs to be carefully considered in the future. The EFs for Hf, Cs, Rb, Th, As, and K in cores ED-2 and ED-3 were lower than 1.5, as shown in Fig. 9. An EF value of less than 1.5 indicates that the content of these elements in the soil is the result of natural processes. The EF of Cl was significantly high, indicating that it was highly enriched in the studied cores. The EF values of Cl ranked the cores under investigation in the following order: ED-3 > ED-2. The marine association is mainly responsible for the high EF of Cl. Furthermore, the Ca EF was significantly higher, and the cores studied were arranged in this manner: ED-3 > ED-2. This may be due to a high biological production rate for the studied cores. According to the literature, when the EF is greater than 1.5, it is possible that metal contamination occurred as a result of anthropogenic activities, such as domestic, industrial, and agricultural effluents, which may contain high amounts of sewage sludge and fertilizers^71,119^. The vertical distribution of EFs demonstrated that values were increasing towards the drain. The analyzed cores were arranged in the following order based on the mean values: ED-3 > ED-2.

Prior to the 1990 s, Edku Lake had the highest EFs of all elements, indicating the effects of early urban and economic growth. Additionally, the Aswan Dam has placed significant pressure on Edku Lake over the decades. Instead of allowing nutrient-rich silt to migrate towards the delta, it has led to its retention. Lake Edku’s brackish water may result from receiving agricultural effluents loaded with fertilizers and pesticides, as well as untreated industrial waste from several factories and effluents from over 300 fish farms via two main drains. Additionally, saline water from El-Boughaz El-Maadiya on the north side contributes to the lake’s brackish water. Therefore, the discharge of untreated wastewater into the lake is responsible for the increasing concentration of heavy metals. To compensate for the lack of nutrients, farmers have increased their use of synthetic fertilizers to support their crops and cope with continued population growth. Between 1965 and 2002, agricultural fertilizer consumption increased from 3.4 × 10^5^ t yr.^−1^ to 13 × 10^5^ t yr.^−1 124^.

**Table 6:**
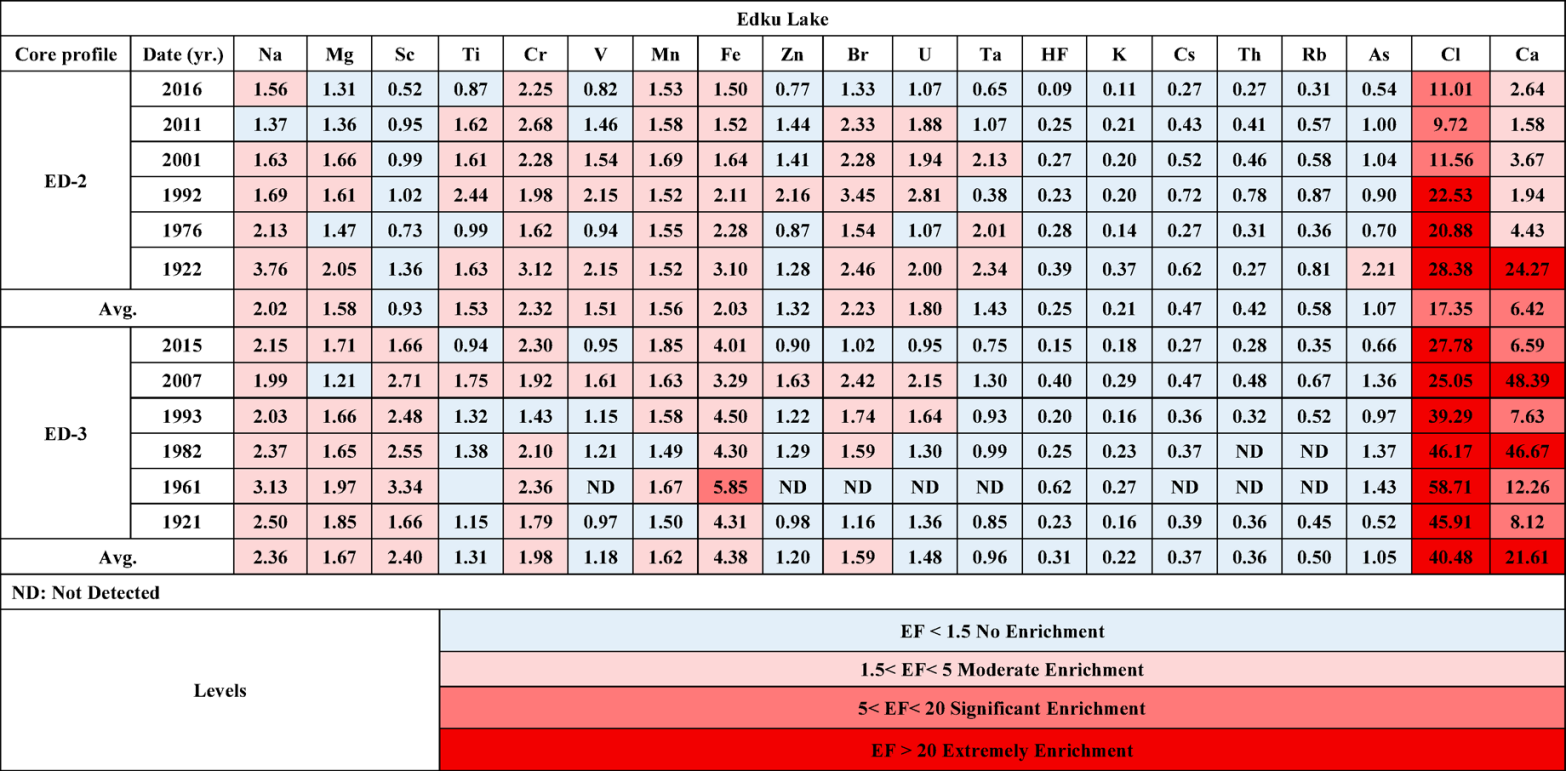
Enrichment factor (EF) values for the core sediments of the studied cores (ED-2, and ED-3). The levels of contamination correspond to the classification of the value range for each index (see^4^).

#### Depositional metal flux (DMF)

**Fig. 10:**
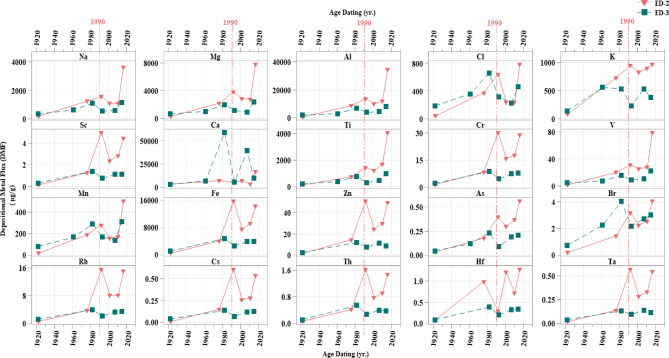
Temporal variations of Depositional Metal Flux (µg/cm^2 year^.) in sediment cores from Edku Lake.

Due to sediment dilution, the metal concentrations in sediments are influenced by the emission factor of the sources and the sedimentation rate at the sampling location^32,121^. Therefore, deposition flux more effectively reflects anthropogenic contributions than concentrations due to the large variation in sediment accumulation rates^122^. The DMF expresses the variation in metal mass over time in a given area and is used to evaluate metal accumulation per unit area over time. The depositional fluxes (µg cm^–2^ yr^–1^) of each metal in the two sediment cores were calculated as shown in Table S3. In cores ED-2 and ED-3, the temporal variability of depositional fluxes exhibited similar patterns, as illustrated in Fig. 10. The sedimentation rate increased from the deeper layers to the upper layers. This indicates that the sedimentation rate of Lake Edku may be accelerating (as mentioned in the section "Sedimentation rates"). This increase can be attributed to the rising flux of metals in the sediments over time, as shown in Fig. 10. The sedimentary fluxes of all metals in Lake Edku have increased, especially since the beginning of the 21 st century. Industrial and agricultural development after the construction of the dams is responsible for the accumulation of metals in the lake. Industrial activities on the coast have increased rapidly due to the economic power generated^46^. However, the calculated metal fluxes in the coastal sediment cores did not provide additional information for reconstructing the aforementioned events, though they can serve as baseline data for temporal mass deposition in the study area.

The DMF values showed a vertical distribution that increased towards the drains; the average value arranged cores in the following order: ED-2 > ED-3. Edku Lake receives a total of 592 × 10⁶ m³/year of untreated drainage water (domestic, agricultural, and industrial) and an additional 348 × 10⁶ m³ from El-Khairy, which connects to three subdrains (Edku, El-Bousily, and Damanhour), as well as the Barsik drain^65^. The drainage water enters the lake from the west and south, then flows northward into the sea. This may be attributed to the higher sedimentation and DMF rates in core ED-2 than in core ED-3.

## Conclusions

The chronology established by the CF-CS and CRS models showed a high degree of congruence in the upper layers of core ED-1. The CIC model’s estimated ages, on the other hand, differed significantly from the other models’ estimates. Based on the findings, the CRS model was chosen, taking into account that Lake Edku receives its caudal flow of precipitation as well as industrial/agricultural drainage water, which implies that the sedimentation rate may have varied due to irregular water release. Accordingly, the CRS model is more appropriate for these lakes as it can account for variations in sedimentation rates over time. The CRS model produced a chronology that was highly correlated with the ^137^Cs peak in the sediment cores analyzed. The mean sedimentation rates (SAR cm/year) predicted by the CRS model showed a general agreement with the CF-CS model and the ^137^Cs time marker estimates in all studied cores except ED-4. A significant increase in accumulation rates was observed across all sediment cores in recent years, a trend potentially driven by anthropogenic activities and shifts in water circulation patterns. The CRS model estimated average sedimentation rates for cores ED−1, ED−2, ED−3, and ED−4 at 4.45 ± 0.15, 6.38 ± 0.37, 1.63 ± 0.11, and 4.42 ± 0.16 cm/yr, respectively. These mean rates for the investigated lake notably exceed those reported for comparable coastal environments.

The k_0_-INAA approach was successfully implemented using a thorough and reliable multi-element analysis focused on evaluating environmental changes in Lake Edku sediment cores and the effect of anthropogenic activities over a 100-year period. The results showed that the elements have different vertical variations in all sediment cores, indicating the presence of different pollution sources in Lake Edku. Furthermore, seawater intrusion may be the cause of the rise in Na, Cl, Br, and Mg concentrations in El-Boughaz as well as in the northern regions of the lake. Additionally, high sodium and magnesium levels were found near drains, possibly as a result of agricultural residues. The increase in bromine (Br) concentration may be due to its use in fertilizers and in the production of fumigants for agricultural use. The amount of calcium in sediment cores was roughly seven to eight times higher than the amount that corresponded to the abundance of continental shale. This may be due to the accumulation of large amounts of rock debris in the sediments. Furthermore, the calcium content of the lake sediments can best be explained by the fact that both the proportion of agricultural activity in the catchment areas of the lakes and the proportion of agricultural wastewater entering the lake have increased. The results of the coefficient of variation (CV%) show that the analyzed elements in core ED–2 exhibit greater variability than those in core ED-3. Most metals showed enrichment factors of more than 1.5, indicating the influence of anthropogenic activities on the metal distribution in the sediments. The observed increase in fluxes of anthropogenic elements in the analyzed cores since the 1990 s is primarily due to runoff, overfishing, increased fish farming and wastewater discharges. The vertical distribution of DMF shows an increase in values in the direction of discharge, with the mean values of the analyzed cores arranged in the following order: ED−2 > ED−3. This can be attributed to a higher sedimentation rate.

The evolution of Lake Edku’s sedimentation dynamics clearly reflects the profound environmental and anthropogenic transformations that have occurred since the construction of the Aswan High Dam. The interruption of the Nile’s natural sediment supply fundamentally altered the lake’s hydrology, sediment composition, and geochemical characteristics. Over time, the dominance of nutrient- and pollutant-rich discharges from agricultural, industrial, and municipal sources has accelerated sedimentation rates, enhanced eutrophication, and intensified ecological degradation. Elemental composition trends further demonstrate the transition from a naturally balanced sedimentary regime controlled by Nile inputs to one heavily influenced by human activities and drainage inflows. These changes highlight the urgent need for integrated management strategies to mitigate pollution, restore water quality, and preserve the ecological stability of Lake Edku.

## Supplementary Information

Below is the link to the electronic supplementary material.

Supplementary Material 1

## Data Availability

All data generated or analyzed during this study are included in this published article.
