# Supplementary material for: 210Pb and 137Cs dating models as tracers of recent sedimentary processes of the shallow lake under anthropogenic activity
Source: Sci Rep. 2026 Mar 28;16:10756. doi: 10.1038/s41598-025-31649-z (PMC13039262; doi:10.1038/s41598-025-31649-z)
Supplement: Supplementary file 1 — Supplementary Material 1 [file 41598_2025_31649_MOESM1_ESM.docx]

**^210^Pb and ^137^Cs dating models as tracers of recent sedimentary processes of the shallow lake under anthropogenic activity**

Noha Imam ^a,*^, Alia Ghanem ^b^, Afaf Nada ^b^, Hosnia Abu-Zeid ^b^, Said A. Shetaia ^c,*^, Waiel E. Madcour ^d^

^a^ Physics & Geology Lab, National Institute of Oceanography and Fisheries (NIOF), Cairo, Egypt.

^b^ Physics Department, Faculty of Women for Arts, Science & Education, Ain Shams University, Cairo, Egypt

^c^ Geology Department, Faculty of Science, Al-Azhar university, Cairo, Egypt

^d^ Radiation Protection department, Nuclear Research Center, Egyptian Atomic Energy Authority, Cairo, Egypt

**Scientific Reports (Sci Rep)**

***Corresponding author**:

-Dr. Noha Abdalla Mohamed Imam,

E-mail: [noha_imam115@hotmail.com](mailto:noha_imam115@hotmail.com)

na.imam@niof.sci.eg

-Dr. Said A. Shetaia,

Geology Department, Faculty of Science, Al-Azhar University, Cairo, Egypt

E-mail: [said­_abdelhady@azhar.edu.eg](mailto:said_abdelhady@azhar.edu.eg)

**Table S1: Sedimentation rates (SAR (cm/yr.) and MAR (gm/cm^2^ yr.)) estimated by CRS and CF-CS ^210^Pb dating models and Cs time marker.**

| **Depth**  **cm** | **ED-1** | | **ED-2** | | **ED-3** | | **ED-4** | |
| --- | --- | --- | --- | --- | --- | --- | --- | --- |
|  | **MAR** | **SAR** | **MAR** | **SAR** | **MAR** | **SAR** | **MAR** | **SAR** |
| **-6.00** | **0.09±0.004** | **2.54±0.13** | **0.20±0.04** | **3.19±0.71** | **0.05±0.01** | **0.99±0.09** | **0.09±0.00** | **2.96±0.16** |
| **-12.00** | **0.10±0.005** | **1.65±0.09** | **0.19±0.03** | **2.35±0.41** | **0.06±0.01** | **1.02±0.11** | **0.07±0.00** | **1.74±0.09** |
| **-18.00** | **0.13±0.01** | **1.25±0.06** | **0.23±0.03** | **2.41±0.36** | **0.08±0.01** | **0.99±0.10** | **0.07±0.00** | **1.31±0.08** |
| **-24.00** | **0.19±0.01** | **1.20±0.06** | **0.21±0.03** | **1.71±0.24** | **0.06±0.01** | **0.67±0.07** | **0.08±0.01** | **1.16±0.08** |
| **-30.00** | **0.27±0.02** | **1.39±0.09** | **0.15±0.02** | **1.12±0.15** | **0.05±0.01** | **0.51±0.06** | **0.10±0.01** | **0.99±0.07** |
| **-36.00** | **0.16±0.01** | **0.95±0.06** | **0.16±0.02** | **1.26±0.17** | **0.07±0.01** | **0.70±0.09** | **0.08±0.01** | **0.64±0.05** |
| **-42.00** | **0.14±0.01** | **0.65±0.04** | **0.17±0.02** | **1.22±0.18** | **0.08±0.01** | **0.73±0.10** | **0.07±0.01** | **0.41±0.03** |
| **-48.00** | **0.19±0.01** | **0.75±0.06** | **0.16±0.02** | **0.92±0.13** | **0.07±0.01** | **0.49±0.07** | **0.04±0.00** | **0.25±0.02** |
| **-54.00** | **0.17±0.01** | **0.58±0.05** | **0.11±0.01** | **0.48±0.06** | **0.06±0.01** | **0.30±0.04** | **0.01±0.00** | **0.13±0.01** |
| **-60.00** | **0.13±0.01** | **0.46±0.05** | **0.07±0.01** | **0.29±0.04** | **0.02±0.00** | **0.11±0.02** |  |  |
| **-66.00** | **0.06±0.01** | **0.24±0.03** | **0.03±0.01** | **0.16±0.03** |  |  |  |  |
| **Average**  **(CRS Model)** | **0.15±0.01** | **1.06±0.06** | **0.14±0.02** | **1.27±0.21** | **0.06±0.01** | **0.65±0.08** | **0.07±0.00** | **1.07±0.07** |
| **CF-CS**  **Model** | **0.13±0.02** | **0.73±0.10** | **0.29±0.03** | **1.99±0.31** | **0.08±0.02** | **1.09±0.21** | **0.09±0.01** | **0.96±0.08** |
| **^137^Cs-Marker** | **1.51** | | **2.04** | | **1.42** | | **1.42** | |

| **Depth (cm)** | **Core**  **ID** | **Na** | **Mg** | **Al** | **Cl** | **K** | **Sc** | **Ca** | **Ti** | **Cr** | **V** | **Mn** | **Fe** | **Zn** | **As** | **Br** | **Rb** | **Cs** | **Th** | **U** | **Ta** | **Hf** |  |
| --- | --- | --- | --- | --- | --- | --- | --- | --- | --- | --- | --- | --- | --- | --- | --- | --- | --- | --- | --- | --- | --- | --- | --- |
| **6** | **ED-2** | **19216**  **±984** | **41157**  **±5958** | **183631±6347** | **4137**  **±502** | **5124**  **±1320** | **23.51**  **±0.67** | **88272**  **±5896** | **21626**  **±1752** | **153.09**  **±4.90** | **415**  **±39** | **2661**  **±247** | **75686**  **±3277** | **262.70**  **±8.67** | **3.00**  **±0.25** | **21.59**  **±0.69** | **79.92**  **±5.97** | **2.86**  **±0.26** | **7.85**  **±0.25** | **3.06**  **±0.43** | **2.89**  **±0.18** | **6.79**  **±0.26** |  |
| **18** |  | **6843**  **±333** | **17215**  **±4098** | **74420**  **±2548** | **1480**  **±302** | **5607**  **±450** | **17.80**  **±0.51** | **21418**  **±1889** | **10430**  **±1154** | **111.26**  **±3.46** | **174**  **±16** | **1093**  **±122** | **57556**  **±2492** | **187.16**  **±6.18** | **2.32**  **±0.18** | **16.15**  **±0.50** | **50.83**  **±4.79** | **1.74**  **±0.19** | **5.77**  **±0.21** | **2.29**  **±0.31** | **2.07**  **±0.10** | **4.53**  **±0.20** |  |
| **30** |  | **7856**  **±585** | **20309**  **±2311** | **71880**  **±2464** | **1700**  **±563** | **5905**  **±517** | **17.10**  **±0.49** | **48022**  **±3059** | **8579**  **±836** | **113.03**  **±3.54** | **179**  **±17** | **1137**  **±110** | **54270**  **±2331** | **177.36**  **±5.85** | **2.16**  **±0.18** | **16.11**  **±0.49** | **58.92**  **±4.16** | **1.89**  **±0.17** | **5.64**  **±0.18** | **2.30**  **±0.30** | **2.06**  **±0.13** | **4.69**  **±0.21** |  |
| **42** |  | **9187**  **±819** | **22224**  **±3402** | **81191**  **±2775** | **3741**  **±525** | **5576**  **±495** | **29.30**  **±0.84** | **28688**  **±2231** | **8399**  **±773** | **178.69**  **±5.66** | **182**  **±17** | **1658**  **±162** | **93948**  **±4067** | **302.82**  **±10.05** | **2.36**  **±0.19** | **18.91**  **±0.89** | **92.59**  **±6.79** | **3.62**  **±0.43** | **9.66**  **±0.30** | **2.23**  **±0.32** | **3.37**  **±0.20** | **7.77**  **±0.29** |  |
| **54** |  | **12394**  **±701** | **21718**  **±3374** | **86928**  **±2983** | **3712**  **±365** | **7269**  **±672** | **12.77**  **±0.37** | **70062**  **±3929** | **7372**  **±1422** | **83.34**  **±2.74** | **199**  **±19** | **1912**  **±161** | **40602**  **±1764** | **144.99**  **±4.81** | **1.80**  **±0.16** | **14.51**  **±0.82** | **37.91**  **±2.87** | **1.52**  **±0.14** | **4.25**  **±0.14** | **1.87**  **±0.26** | **1.38**  **±0.10** | **3.92**  **±0.15** |  |
| **66** |  | **5626**  **±350** | **7789**  **±1520** | **22310**  **±794** | **1295**  **±156** | **2637**  **±183** | **5.38**  **±0.15** | **98441**  **±4963** | **3641**  **±436** | **49.04**  **±1.50** | **50**  **±5** | **668**  **±63** | **15264**  **±667** | **59.21**  **±2.24** | **1.22**  **±0.08** | **6.91**  **±0.40** | **21.87**  **±2.09** | **0.34**  **±0.16** | **2.47**  **±0.08** | **1.51**  **±0.19** | **0.66**  **±0.06** | **2.97**  **±0.13** |  |
| **Mean± E** | | **10187**  **±629** | **21735**  **±3443** | **86727**  **±2985** | **2678**  **±402** | **5353**  **±606** | **17.64**  **±0.51** | **59151**  **±3661** | **10009**  **±1062** | **115**  **±3.63** | **200**  **±19** | **1522**  **±144** | **56221**  **±2433** | **189**  **±6.30** | **2.14**  **±0.17** | **15.70**  **±0.63** | **57.01**  **±4.45** | **2.00**  **±0.23** | **5.94**  **±0.17** | **2.21**  **±0.30** | **2.07**  **±0.13** | **5.78**  **±0.21** |  |
| **CV%** | | **49** | **50** | **61** | **49** | **28** | **47** | **53** | **61** | **41** | **59** | **47** | **49** | **46** | **28** | **32** | **46** | **57** | **43** | **23** | **47** | **56** |  |
| **6** | **ED-3** | **14448**  **±812** | **29272**  **±3369** | **100457**  **±3459** | **5709**  **±958** | **4668**  **±619** | **14.02**  **±0.40** | **120411±6355** | **12075**  **±888** | **97.85**  **±3.18** | **274**  **±26** | **3889**  **±296** | **48348**  **±2091** | **111.06**  **±4.20** | **2.62**  **±0.28** | **37.86**  **±1.51** | **43.78**  **±3.65** | **1.58**  **±0.17** | **4.84**  **±0.17** | **2.02**  **±0.36** | **1.41**  **±0.13** | **4.28**  **±0.18** |  |
| **18** |  | **8449**  **±518** | **13001**  **±5783** | **63264**  **±2173** | **3241**  **±674** | **7607**  **±1409** | **16.34**  **±0.47** | **55659**  **±3546** | **6345**  **±948** | **104.32**  **±3.52** | **152**  **±15** | **2011**  **±173** | **55278**  **±2391** | **165.44**  **±5.64** | **2.75**  **±0.30** | **39.01**  **±1.70** | **47.42**  **±5.36** | **1.74**  **±0.21** | **5.82**  **±0.19** | **2.64**  **±0.39** | **2.01**  **±0.14** | **4.67**  **±0.18** |  |
| **30** |  | **13262**  **±1203** | **27553**  **±4231** | **97534**  **±3337** | **7838**  **±846** | **5782**  **±1163** | **19.05**  **±0.55** | **135228±6637** | **7281**  **±1237** | **115.16**  **±3.62** | **227**  **±21** | **4241**  **±345** | **63816**  **±2766** | **183.12**  **±6.18** | **2.35**  **±0.33** | **55.00**  **±2.00** | **55.13**  **±4.24** | **1.75**  **±0.22** | **6.94**  **±0.22** | **2.91**  **±0.42** | **2.37**  **±0.14** | **5.15**  **±0.22** |  |
| **42** |  | **15731**  **±1080** | **27845**  **±4219** | **99166**  **±3399** | **9365**  **±674** | **7577**  **±1247** | **20.28**  **±0.58** | **84146**  **±4713** | **10893**  **±1039** | **122.87**  **±4.34** | **219**  **±21** | **4117**  **±283** | **68355**  **±2958** | **170.22**  **±5.87** | **3.33±0.35** | **57.47**  **±2.23** | **57.63**  **±3.50** | **1.98**  **±0.13** | **7.85**  **±0.29** | **4.16**  **±0.42** | **1.90**  **±0.11** | **5.55**  **±0.21** |  |
| **54** |  | **15569**  **±1022** | **24906**  **±3670** | **74162**  **±2539** | **8906**  **±726** | **13826**  **±2749** | **ND** | **165341**  **±7476** | **9158**  **±927** | **ND** | **183**  **±17** | **4193**  **±283** | **ND** | **ND** | **3.00**  **±0.50** | **56.36**  **±2.22** | **ND** | **ND** | **ND** | **3.26**  **±0.43** | **ND** | **ND** |  |
| **66** |  | **17969**  **±1045** | **33728**  **±3889** | **107068±3674** | **10055**  **±806** | **7359**  **±1266** | **18.17**  **±0.52** | **158088**  **±7633** | **10020**  **±1224** | **105.95**  **±3.44** | **237**  **±23** | **4461**  **±290** | **56178**  **±2438** | **133.64**  **±4.70** | **2.57**  **±0.33** | **40.36**  **±1.66** | **66.87**  **±3.31** | **2.22**  **±0.20** | **6.55**  **±0.20** | **1.72**  **±0.39** | **2.15**  **±0.17** | **5.16**  **±0.20** |  |
| **Mean± E** | | **14238**  **±947** | **26051**  **±4193** | **90276**  **±3097** | **7519**  **±780** | **7803**  **±1408** | **17.57**  **±0.64** | **329502±6060** | **9295.33±1044** | **109**  **±3.74** | **215**  **±21** | **3819**  **±279** | **58395**  **±2528** | **153**  **±5.00** | **2.77**  **±0.14** | **47.68**  **±1.70** | **54.17**  **±4.01** | **1.85**  **±0.00** | **6.40**  **±0.94** | **2.79**  **±0.21** | **1.97**  **±0.07** | **4.96**  **±0.78** |  |
| **CV%** | | **23** | **27** | **19** | **34** | **41** | **14** | **91** | **23** | **9** | **20** | **24** | **13** | **19** | **13** | **20** | **17** | **13** | **18** | **32** | **18** | **10** |  |
| **ND: Not Detected**  **CV%: Coefficient of Variation**  **E: Error** | | | | | | | | | | | | | | | | | | | | | | | |

**Table S2: Metal concentrations (μg/g) and Coefficient of Variation (CV%) in the selected sediment core samples of Edku Lake**

**Table S3: Depositional Metal Flux (µg/cm^2^ yr.) in cores of Edku Lake (ED-2 and ED-3).**

| **Core profile** | **Date (yr.)** | **MAR**  **g cm^-2^ yr.^-1^** | **Na** | **Mg** | **Al** | **Cl** | **K** | **Sc** | **Ca** | **Ti** | **Cr** | **V** | **Mn** | **Fe** | **Zn** | **As** | **Br** | **Rb** | **Cs** | **Th** | **U** | **Ta** | **Hf** |
| --- | --- | --- | --- | --- | --- | --- | --- | --- | --- | --- | --- | --- | --- | --- | --- | --- | --- | --- | --- | --- | --- | --- | --- |
| **ED-2** | **2016** | **0.20** | **3900** | **8354** | **37272** | **840** | **1040** | **4.77** | **17917** | **4390** | **31.07** | **84.23** | **540** | **15362** | **53.32** | **0.61** | **4.38** | **16.22** | **0.58** | **1.59** | **0.62** | **0.59** | **1.38** |
|  | **2011** | **0.23** | **1551** | **3902** | **16870** | **336** | **1271** | **4.04** | **4855** | **2364** | **25.22** | **39.44** | **248** | **13047** | **42.41** | **0.53** | **3.66** | **11.52** | **0.39** | **1.31** | **0.52** | **0.47** | **1.03** |
|  | **2003** | **0.15** | **1168** | **3019** | **10684** | **253** | **878** | **2.54** | **7138** | **1275** | **16.80** | **26.61** | **169** | **8067** | **26.35** | **0.32** | **2.39** | **8.76** | **0.28** | **0.84** | **0.34** | **0.31** | **1.29** |
|  | **1994** | **0.17** | **1589** | **3844** | **14044** | **647** | **965** | **5.07** | **4962** | **1453** | **30.91** | **31.48** | **287** | **16251** | **52.38** | **0.41** | **3.27** | **16.02** | **0.63** | **1.67** | **0.39** | **0.58** | **0.31** |
|  | **1979** | **0.11** | **1302** | **2282** | **9134** | **390** | **764** | **1.34** | **7361** | **775** | **8.76** | **20.91** | **201** | **4266** | **15.22** | **0.19** | **1.52** | **3.98** | **0.16** | **0.45** | **0.20** | **0.14** | **1.04** |
|  | **1932** | **0.03** | **195** | **270** | **774** | **45** | **91** | **0.19** | **3414** | **126** | **1.70** | **1.73** | **23** | **529** | **2.05** | **0.04** | **0.24** | **0.76** | **0.01** | **0.09** | **0.05** | **0.02** | **0.10** |
| **Avg.** | | **0.15** | **1469** | **3267** | **13337** | **384** | **742** | **2.69** | **6960** | **1542** | **17.15** | **30.68** | **222.13** | **8613** | **28.74** | **0.31** | **2.30** | **8.63** | **0.31** | **0.89** | **0.31** | **0.32** | **0.77** |
| **Core profile** | **Date (yr.)** | **MAR**  **g cm^-2^ yr.^-1^** | **Na** | **Mg** | **Al** | **Cl** | **K** | **Sc** | **Ca** | **Ti** | **Cr** | **V** | **Mn** | **Fe** | **Zn** | **As** | **Br** | **Rb** | **Cs** | **Th** | **U** | **Ta** | **Hf** |
| **ED-3** | **2014** | **0.05** | **786** | **1593** | **5468** | **311** | **254** | **0.76** | **6556** | **657** | **5.33** | **14.92** | **211.70** | **2632** | **6.05** | **0.14** | **2.06** | **2.38** | **0.09** | **0.26** | **0.11** | **0.08** | **0.23** |
|  | **2006** | **0.08** | **681** | **1047** | **5096** | **261** | **613** | **1.32** | **44834** | **511** | **8.40** | **12.24** | **162.00** | **4453** | **13.33** | **0.22** | **3.14** | **3.82** | **0.14** | **0.47** | **0.21** | **0.16** | **0.38** |
|  | **1994** | **0.05** | **665** | **1382** | **4891** | **393** | **290** | **0.96** | **6781** | **365** | **5.78** | **11.38** | **212.69** | **3200** | **9.18** | **0.12** | **2.76** | **2.76** | **0.09** | **0.35** | **0.15** | **0.12** | **0.26** |
|  | **1983** | **0.08** | **1323** | **2341** | **8337** | **787** | **637** | **1.70** | **70741** | **916** | **10.33** | **18.41** | **346.12** | **5747** | **14.31** | **0.28** | **4.83** | **4.85** | **0.17** | **0.66** | **0.35** | **0.16** | **0.47** |
|  | **1973** | **0.06** | **884** | **1415** | **4213** | **506** | **785** | **0.00** | **9392** | **520** | **ND** | **10.40** | **238.19** | **ND** | **ND** | **0.17** | **3.20** | **ND** | **ND** | **ND** | **0.19** | **ND** | **ND** |
|  | **1909** | **0.04** | **719** | **1349** | **4283** | **402** | **294** | **0.73** | **6320** | **401** | **4.24** | **9.48** | **178.44** | **2247** | **5.35** | **0.10** | **1.61** | **2.67** | **0.09** | **0.26** | **0.07** | **0.09** | **0.21** |
| **Avg.** | | **0.06** | **843** | **1521** | **5381** | **443** | **479** | **0.91** | **24104** | **562** | **6.81** | **12.81** | **224.86** | **3656** | **9.64** | **0.17** | **2.93** | **3.30** | **0.11** | **0.40** | **0.18** | **0.12** | **0.31** |
| **ND: Not Detected** | | | | | | | | | | | | | | | | | | | | | | | |
